# A Claudin-9–Based Ion Permeability Barrier Is Essential for Hearing

**DOI:** 10.1371/journal.pgen.1000610

**Published:** 2009-08-21

**Authors:** Yoko Nakano, Sung H. Kim, Hyoung-Mi Kim, Joel D. Sanneman, Yuzhou Zhang, Richard J. H. Smith, Daniel C. Marcus, Philine Wangemann, Randy A. Nessler, Botond Bánfi

**Affiliations:** 1 Department of Anatomy and Cell Biology, University of Iowa, Iowa City, Iowa, United States of America; 2 Inflammation Program, University of Iowa, Coralville, Iowa, United States of America; 3 Department of Anatomy and Physiology, Kansas State University, Manhattan, Kansas, United States of America; 4 Department of Otolaryngology – Head and Neck Surgery, University of Iowa, Iowa City, Iowa, United States of America; 5 Central Microscopy Research Facility, University of Iowa, Iowa City, Iowa, United States of America; 6 Department of Internal Medicine, University of Iowa, Iowa City, Iowa, United States of America; National Institute on Deafness and Other Communication Disorders, National Institutes of Health, United States of America

## Abstract

Hereditary hearing loss is one of the most common birth defects, yet the majority of genes required for audition is thought to remain unidentified. Ethylnitrosourea (ENU)–mutagenesis has been a valuable approach for generating new animal models of deafness and discovering previously unrecognized gene functions. Here we report on the characterization of a new ENU–induced mouse mutant (nmf329) that exhibits recessively inherited deafness. We found a widespread loss of sensory hair cells in the hearing organs of nmf329 mice after the second week of life. Positional cloning revealed that the nmf329 strain carries a missense mutation in the *claudin-9* gene, which encodes a tight junction protein with unknown biological function. In an epithelial cell line, heterologous expression of wild-type claudin-9 reduced the paracellular permeability to Na^+^ and K^+^, and the *nmf329* mutation eliminated this ion barrier function without affecting the plasma membrane localization of claudin-9. In the nmf329 mouse line, the perilymphatic K^+^ concentration was found to be elevated, suggesting that the cochlear tight junctions were dysfunctional. Furthermore, the hair-cell loss in the claudin-9–defective cochlea was rescued *in vitro* when the explanted hearing organs were cultured in a low-K^+^ milieu and *in vivo* when the endocochlear K^+^-driving force was diminished by deletion of the *pou3f4* gene. Overall, our data indicate that claudin-9 is required for the preservation of sensory cells in the hearing organ because claudin-9–defective tight junctions fail to shield the basolateral side of hair cells from the K^+^-rich endolymph. In the tight-junction complexes of hair cells, claudin-9 is localized specifically to a subdomain that is underneath more apical tight-junction strands formed by other claudins. Thus, the analysis of *claudin-9* mutant mice suggests that even the deeper (subapical) tight-junction strands have biologically important ion barrier function.

## Author Summary

Hereditary deafness is a common birth defect in the human population, yet the majority of genes required for audition is thought to be unidentified. Genetic approaches in the mouse have greatly contributed to our understanding of the molecular mechanisms that underlie hearing. Random mutagenesis of mice, identification of deaf mutants, and subsequent analysis of the deafness-causing gene defects has led to the discovery of several previously unrecognized gene functions. Here, we report on the characterization of a new mutant mouse line (nmf329) that exhibits profound hearing loss and loss of sensory cells in the auditory organ. Genetic analysis reveals that these animals carry a mutation in the *claudin-9* gene, which encodes a protein with hitherto unknown biological function. We have found that normal claudin-9—but not a mutant form—inhibits the paracellular movement of certain ions. The lack of the claudin-9 ion barrier in the inner ear leads to changes in ionic conditions that can account for the loss of sensory cells in the mutant mice. Within the cell–cell junctions, the claudin-9 layer is located basal to those of other claudins. Thus, our analysis of claudin-9–deficient animals suggests that even the deeper layers of claudins have important ion barrier function.

## Introduction

The separation of compositionally distinct extracellular fluids is a vital function of the epithelial and endothelial cells that line the lumens of organs and blood vessels, respectively. Indiscriminate diffusion of solutes across epithelial and endothelial sheets is prevented by sealing of the paracellular spaces by tight junctions [1],[2]. These intercellular junctions are comprised of membrane-associated and transmembrane proteins that form continuous networks of strands circumscribing the apical end of the cell. The tight-junction strands of neighboring cells are arranged in parallel fashion as they associate with each other laterally, thereby creating paracellular barriers to the diffusion of solutes [3]. In addition to forming barriers, tight junctions also function as signaling hubs, and contribute to the maintenance of cell polarity [4],[5].

The “tightness” of tight junctions varies greatly from tissue to tissue, and depends largely on the presence of integral membrane proteins known as claudins [6]. Twenty-four claudins have been identified in humans. Each of these is a four-pass transmembrane protein with intracellular N- and C-termini and two extracellular loops. The first extracellular loop is responsible for the selective ion barrier function of the claudin [7]–[10], and is also critical for the polymerization of neighboring claudins into strands [11]. The second extracellular loop is thought to be important for the intercellular claudin-claudin interactions [12]. The claudin C-terminus binds to scaffolding proteins such as zonula occludens-1, -2, -3 and MUPP-1, which link the tight junction to the actin cytoskeleton [13]–[17].

The expression of individual claudins can have diverse effects on the paracellular permeability of solutes. Some claudins create paracellular ion barriers [9], [18]–[22], whereas others form ion-selective paracellular channels [8], [18], [23]–[26]. The properties of tight junctions are further diversified by the expression of multiple claudins in most epithelial cells. Moreover, some (but not all) claudins can interact with other members of the claudin family, either within or between the tight junction strands, and this affects paracellular ion permeability [27]. Thus, the ion barrier property of epithelial and endothelial cell layers depends on the type and ratio of different claudins that are present, and also on the interactions among them [28].

**Figure 1:**
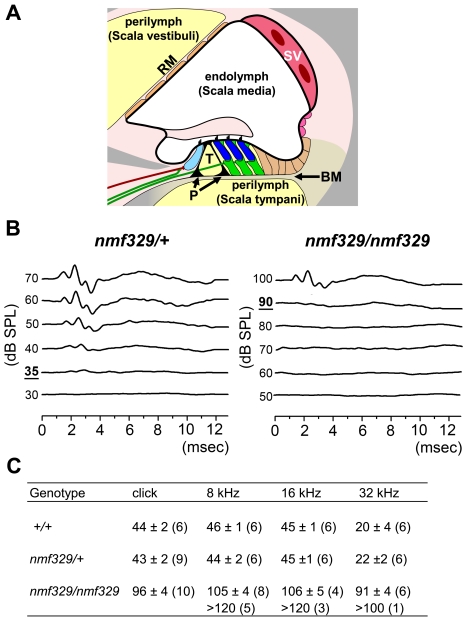
Deafness in nmf329 mice. (A) Schematic diagram of a cochlear turn. The *scala vestibuli* and *scala tympani* are filled with perilymph (yellow), whereas the *scala media* is filled with endolymph. The thick black line indicates the tight junction barrier, which insulates the endolymphatic compartment and both sides of the *stria vascularis* (SV). The organ of Corti contains one row of inner hair cells (light blue), three rows of outer hair cells (dark blue), three rows of Deiters' supporting cells (green), and two rows of pillar cells (P) that form the tunnel of Corti (T). The paracellular spaces in the organ of Corti are filled with basolateral fluid (yellow shading), and this fluid communicates with the perilymph through the basilar membrane (BM). RM indicates the Reissner's membrane. (B) Representative ABR waveforms for *nmf329/+* mice and *nmf329/nmf329* mice at P28. Broadband click stimuli were applied at the indicated intensities, in dB-SPL. Bold and underlined numbers indicate the auditory thresholds. (C) Mean ABR thresholds (±SEM) to broadband click stimuli and pure-tone sounds in wild-type (+/+), *nmf329/+*, and *nmf329/nmf329* mice at P28. Mice with ABR thresholds higher than the maximum SPL (120 dB or 100 dB, depending on the frequency) were not included in the statistical analysis, and are listed separately (bottom row). The number of mice used for the generation of ABR data is shown in parenthesis.

The hearing organ provides a unique example of the need to separate compositionally distinct extracellular fluids. The stereocilia of the cochlear sensory hair cells are surrounded by the endolymph, in which the K^+^ concentration is high (∼140 mM) and the Na^+^ concentration is low (∼1 mM). In contrast, the basolateral side of hair cells is bathed in the perilymph, which has low K^+^ and high Na^+^ concentrations (∼2 mM and ∼140 mM, respectively). These ion gradients are generated by the *stria vascularis* (Figure 1A), which also produces an endocochlear potential (EP, ∼80 mV) between the endolymphatic and perilymphatic spaces [29]. The EP amplifies the K^+^ driving force. In this context, sound-induced deflections of stereocilia result in the entry of K^+^ into the hair cell through apical ion channels. The ensuing depolarization of the cell leads to sound detection. Subsequently, the resting potential of the hair cell is restored by K^+^ efflux from the basolateral side of the cell [30]. The K^+^ and voltage gradients between the apical and basolateral sides of hair cells are sustained by tightly sealed epithelial cells that line the entire endolymphatic compartment and both sides of the *stria vascularis* (Figure 1A) [31]–[33].

Of all the claudin family members, claudin-9 is the most highly expressed in the inner ear [34], and it is present in all of the major epithelial cell types that line the endolymphatic space [35]. However, the biological function of claudin-9 has not been established. Here we report the molecular and phenotypic characterization of a previously unrecognized claudin-9-deficient mouse strain, nmf329, which was generated in the course of an ENU-induced mutagenesis project at The Jackson Laboratory [36]. Our analysis of nmf329 mice indicates that the genetic defect in *claudin-9* leads to deafness, and that a claudin-9-based ion barrier is necessary to protect the basolateral domain of the hair cell from the K^+^-rich endolymph.

## Results

### Non-syndromic deafness in the nmf329 mouse line

Mice of the nmf329 line were tested for their reactions to loud sound bursts generated by a click box (∼90 dB sound pressure level, SPL). Whereas wild-type mice exhibited typical startle responses, nmf329 homozygotes did not react. To evaluate the hearing loss of the nmf329 mice more quantitatively, we measured the auditory brainstem response (ABR) of these animals on postnatal day 28 (P28), using broadband and pure-tone sounds. We found that the ABR threshold of *nmf329/nmf329* mice was elevated by ∼60 dB-SPL compared to that of wild-type littermates, whereas the ABR thresholds of heterozygous and wild-type animals were indistinguishable from each other (Figure 1B and 1C). The hearing loss of *nmf329/nmf329* mice was equally severe at high- and low-frequency, as indicated by the uniformly high ABR thresholds at 8, 16, and 32 kHz (Figure 1C). Next, we investigated whether *nmf329/nmf329* mice develop hearing for a brief period prior to P28. We found that the hearing loss of homozygous mutant mice was already severe at P16 (i.e. 3–4 days after the expected onset of hearing, Figure S1). These results indicate that the deafness caused by the *nmf329* mutation has an early onset.

Given that auditory defects are often accompanied by problems with balance, we quantified the balancing abilities of nmf329 mice by measuring the time that they could remain on a slowly rotating rod. Equally good balance was demonstrated by wild-type, *nmf329/+*, and *nmf329/nmf329* mice (Figure S2). Also, intercrosses between heterozygotes produced deaf progeny at a frequency of 28%, suggesting that *nmf329/nmf329* mice are born at the Mendelian ratio (i.e. 25%), and that the *nmf329* mutation does not lead to embryonic lethality. Histopathological examination of the heart, lung, ileum, colon, liver, and kidney for other defects did not reveal abnormalities in the *nmf329/nmf329* mice (data not shown). Overall, these results indicate that the *nmf329* mutation leads to recessively inherited non-syndromic deafness.

### Cochlear hair-cell degeneration in the nmf329 mouse line

**Figure 2:**
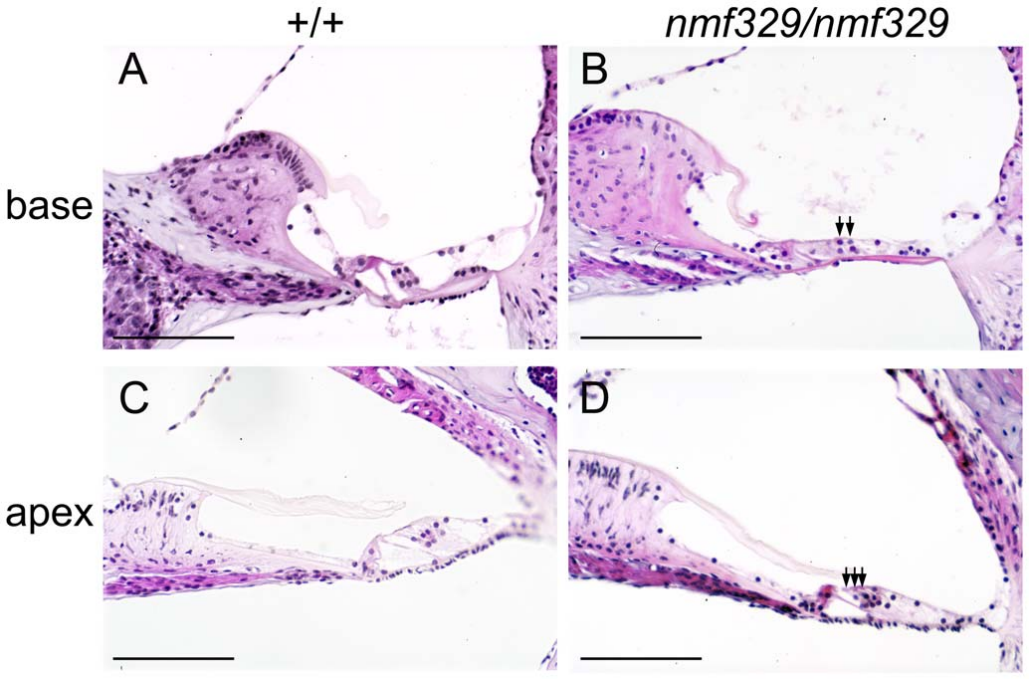
Morphological defects at the basal turn of the cochlea in the nmf329 mouse line. (A–D) Hematoxylin- and eosin-stained cross sections of cochleas from +/+ and *nmf329/nmf329* mice (P28), at the basal turn (base) and apical turn (apex). In contrast to the intact cochlear morphology of a control mouse (A), the basal cochlear turn of an *nmf329/nmf329* mouse lacks the tunnel of Corti and contains only two rows of OHCs (B). The apical turn of the cochlea is intact in both the +/+ and *nmf329/nmf329* mice (C, D). Arrows indicate the OHCs in the *nmf329/nmf329* sample. Scale bars: 100 µm.

Histological analysis of cross-sectioned cochleas from *nmf329/nmf329* mice (P28) demonstrated that the overall structure of the hearing organ was maintained in the nmf329 line, and that the *stria vascularis*, Reissner's membrane, and spiral ganglion neurons were morphologically normal (Figure S3). Furthermore, no signs of inflammation were detected in the hearing organ (Figure S4). However, the organ of Corti was deformed at the basal turn of the *nmf329/nmf329* cochlea; it collapsed into a compact cell layer that lacked paracellular spaces, and it contained only two rows of outer hair cells (OHCs, Figure 2B). This malformation was not present along the entire length of the hearing organ; at the apical turn, the organ of Corti appeared intact, with three rows of OHCs present (Figure 2D). In contrast, cochlear sections from *nmf329/+* mice (data not shown) and wild-type mice (Figure 2A and 2C) exhibited normal morphology both at the basal and apical turns. These data indicate that the *nmf329* mutation leads to a loss of OHCs at the basal turn of the cochlea by P28.

**Figure 3:**
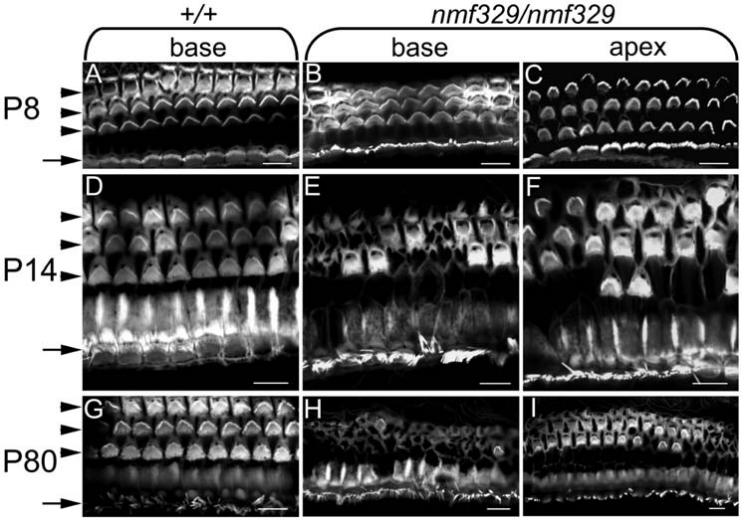
Degeneration of OHCs in the cochleas of nmf329 mice after P8. (A–I) Organ of Corti preparations from +/+ and *nmf329/nmf329* mice were stained with phalloidin-Alexa Fluor 488 to visualize actin-rich structures including the stereocilia. At P8, the bundles of stereocilia are present in all three rows of OHCs (arrowheads) and in the row of IHCs (arrow) in the cochleas of both +/+ (A) and *nmf329/nmf329* mice (B, C). At P14, the basal turn of the +/+ cochlea contains three intact rows of OHCs (D), whereas the corresponding region of the nmf329 cochlea lacks the majority of stereociliary bundles (E). Where the cell-cell junctions are in focus, large polygonal cells can be seen to replace the missing OHCs (center of panel E). At the apical turn of the same cochlea, a few stereociliary bundles are missing from the first row of OHCs (F). At P80, the control cochlea is undamaged (G), but the *nmf329/nmf329* cochlea contains only a small number of OHCs at the basal turn, and the majority of OHCs are replaced by non-ciliated cells (H). At the apical turn of the nmf329 cochlea (P80), several OHCs are replaced by large polygonal cells, especially in the first row (I). Scale bars: 10 µm.

To assess the extent of the hair-cell loss further, organ of Corti samples were dissected from wild-type, *nmf329/+*, and *nmf329/nmf329* mice at various stages of development, and stereociliary bundles and other actin-rich structures were visualized using fluorescently labeled phalloidin. In young *nmf329/nmf329* animals (P8), the stereociliary bundles of IHCs and all three rows of OHCs were present and intact (Figure 3B and 3C), suggesting that the initial steps of hair-cell development were unaffected by the *nmf329* allele. However, by P14, widespread degeneration was observed in the organ of Corti in the homozygous mutant mice. Specifically, at the basal turn, most OHC stereociliary bundles were missing, especially from the first and second rows of OHCs (Figure 3E; low magnification images and statistical analyses are shown in Figure S5, S6, S7). At the apex of the cochlea, the defect was milder: approximately half of the stereociliary bundles were missing from the first OHC row, whereas the second and third OHC rows were largely intact (Figure 3F, Figure S5, Figure S7). Images focused at the level of cell-cell junctions showed that many of the OHCs were missing from the reticular lamina and replaced by large polygonal cells (Figure 3E and 3F, Figure S6). The loss of OHCs from the reticular lamina was confirmed by visualizing cell-cell junctions in the organ of Corti with an anti-occludin antibody (Figure S8). In contrast to the widespread loss of OHCs, the IHCs were preserved even at the basal turn of the cochlea in *nmf329/nmf329* mice (Figure 3H and Figure S6). Surprisingly, our analysis of older *nmf329/nmf329* mice revealed that the initially rapid degeneration of the hearing organ slowed down after P14; at P80, the basal turn of cochlea still contained a few OHCs (Figure 3H; Figure S5, S6, S7). At the cochlear apex, the loss of hair cells was particularly apparent in the first row of OHCs (Figure 3I; Figure S5, S6, S7). In contrast to the homozygous mutants, both wild-type mice and *nmf329/+* mice had three intact rows of OHCs at each time point investigated (Figure 3A, 3D, and 3G). Overall, these data demonstrate a widespread loss of OHCs in the *nmf329/nmf329* mice after P8. Furthermore, the cochlear defect in the mutant mice was morphologically similar to the previously described “phalangeal scars”, which are formed by supporting cells when OHCs are lost due to a variety of causes, such as noise trauma, ototoxic aminoglycosides, and aging [37],[38].

### Mapping and identification of the *nmf329* allele

**Figure 4:**
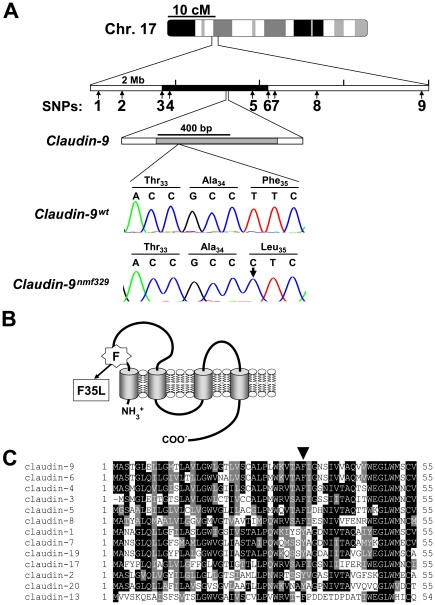
Missense mutation in the *claudin-9* gene of nmf329 mice. (A) Nine SNPs were used to refine the position of *nmf329* to a ∼2.5-megabase region on chromosome 17, between SNP-3 (rs32299331) and SNP-6 (rs33167092). The nmf329 linkage region includes the *claudin-9* gene, which contains only one exon (gray box). A deoxythymidine nucleotide (T) of wild-type c*laudin-9* (upper chromatogram) is replaced by a deoxycytosine (C) in the *nmf329* mutant strain, as indicated by an arrow in the lower chromatogram. The point mutation changes the 35th amino acid of claudin-9 from phenylalanine (Phe) to leucine (Leu), as shown in the translation lines. (B) The phenylalanine-to-leucine amino acid substitution (F35L) is localized to the first predicted extracellular loop of claudin-9. (C) Alignment of claudin-9 and its 12 closest paralogs found in the mouse genome. Black shading indicates residue identity; gray shading indicates aminoacyl group similarity. The arrowhead marks the phenylalanine residue of claudin-9 that is replaced with leucine in the nmf329 mice.

The Jackson Laboratory had previously mapped the mutation in the nmf329 mouse line to chromosome 17, ∼9.6 cM from the centromere [36]. Since this linkage-map position implicated several hundred genes as potential candidates for *nmf329*, we refined the location of the *nmf329* allele to a physical interval on chromosome 17. *Nmf329/nmf329* mice (on the C57Bl/6 background) were outcrossed to the A/J strain, and the F1 offspring were then backcrossed to the nmf329 line. Progeny of this backcross (116 mice) were assessed for hearing by ABR measurement, and analyzed for crossovers by SNP detection over an 8-megabase genomic segment (see Figure 4A). This approach refined the position of the *nmf329* locus to a 2.5-megabase region between the SNPs rs33167092 and rs32299331. The identified genomic interval contains 76 annotated genes, none of which had been associated with deafness. Therefore, we selected 3 candidate genes (*claudin-9*, *claudin-6*, and *Kctd5*) based on their detection in cochlear cDNA libraries (Unigene database information, NCBI). Sequence analysis of the candidate genes from the nmf329 line revealed that one of them, *claudin-9*, contained a T to C point mutation 102 bases downstream of the start codon (Figure 4A). The c.102T→C alteration led to a phenylalanine (F) to leucine (L) substitution in the first predicted extracellular loop of the encoded protein (Figure 4B). The affected amino acid (F35) is conserved among all claudin-9 orthologs that have been identified thus far (Figure S9). Moreover, F35 is conserved amongst many members of the claudin protein family (Figure 4C). These results suggest that the *claudin-9* mutation in the nmf329 mice may have functional consequences.

### Opposing effects of wild-type and mutant claudin-9 on transepithelial resistance

**Figure 5:**
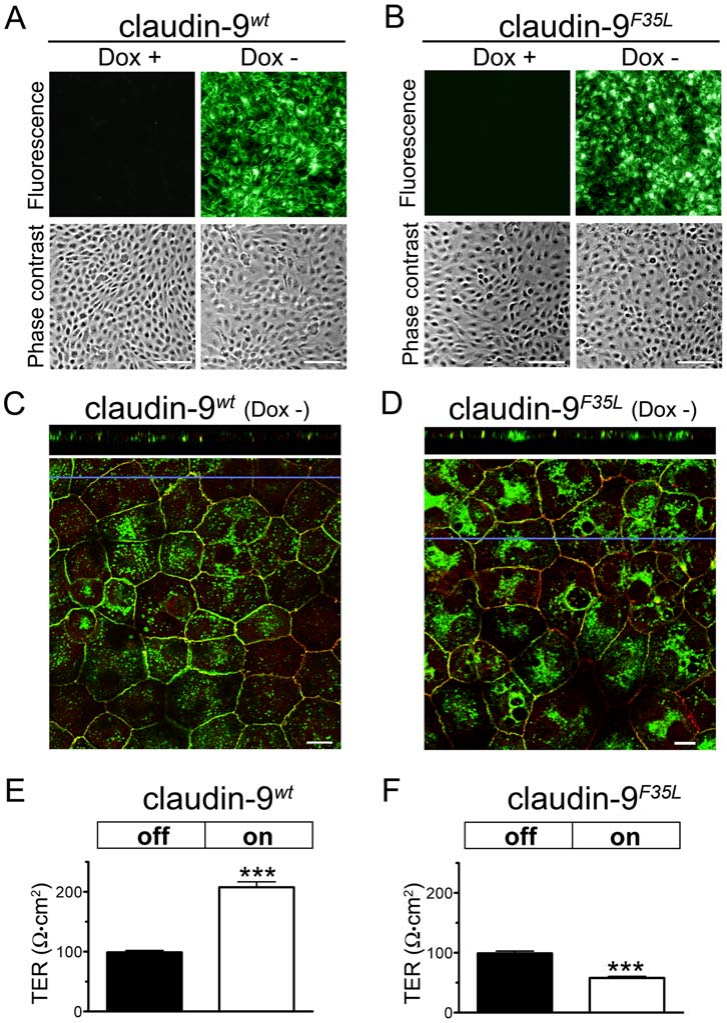
Heterologous expression of wild-type and mutant claudin-9 in MDCK cells alters transepithelial resistance. Stable clones of Tet-off MDCK cells transfected with (A) EYFP-claudin-9*^wt^* and (B) EYFP-claudin-9*^F35L^* fusion constructs. These MDCK cell clones were cultured in the presence and absence of doxycycline (Dox), to turn EYFP-claudin-9 expression “off” and “on”, respectively. Fluorescence and phase-contrast images show that doxycycline control of EYFP-claudin-9 expression is effective. Scale bars: 100 µm. (C, D) Confocal *X-Y* sections through the apical plane of MDCK cells expressing (C) EYFP-claudin-9*^wt^* (green pseudocolor) and (D) EYFP-claudin-9*^F35L^* (green pseudocolor). Anti-occludin immunostaining (red) was used to label the tight junctions of MDCK cells. Yellow color indicates co-localization of the claudin-9 fusion proteins with occludin. Horizontal blue lines indicate the position of *Z* stack slices that are shown in the upper panels. Scale bars: 10 µm. (E, F) The transepithelial resistance (TER) of EYFP-claudin-9*^wt^* and EYFP-claudin-9*^F35L^* transfected Tet-off MDCK cell clones was measured after turning heterologous gene expression “off” (black bars) or “on” (white bars). (E) Induction of EYFP-claudin-9*^wt^* expression increased the TER of the MDCK monolayers (mean±SEM, n = 3 in each of the 3 tested clones, paired *t* test, ***p<0.0001). (F) In contrast, induction of EYFP-claudin-9*^F35L^* expression reduced the TER of the MDCK monolayers (mean±SEM, n = 4 in each of the 3 tested clones, paired *t* test, ***p<0.0001).

To explore the molecular function of the wild-type and mutant claudin-9 proteins (claudin-9*^wt^* and claudin-9*^F35L^*, respectively), we examined the potential electrophysiological consequences of claudin-9*^wt^* and claudin-9*^F35L^* expression in an epithelial cell line. Since N-terminal tags do not interfere with the function of claudins but facilitate expression analysis [20], we fused the N-termini of claudin-9*^wt^* and claudin-9*^F35L^* to EYFP. The DNA sequences of these fusion constructs were inserted downstream of a tetracycline response element and transfected into Tet-off MDCK cells, which do not contain detectable claudin-9 mRNA (data not shown). Stable clones of transfected Tet-off MDCK cells were isolated and subsequently cultured in the presence or absence of doxycycline. Fluorescence microscopy demonstrated that doxycycline treatment “turned off” the expression of both EYFP-claudin-9*^wt^* and EYFP-claudin-9*^F35L^*, whereas the lack of doxycycline resulted in expression of transfected proteins in virtually all cells (Figure 5A and 5B). Furthermore, both EYFP-claudin-9*^wt^* and EYFP-claudin-9*^F35L^* were co-localized with the tight-junction protein occludin in the plasma membrane, indicating that the F35L alteration does not prevent the localization of claudin-9 to tight junctions (Figure 5C and 5D; the EYFP and occludin signals are shown individually in Figure S10).

To evaluate the effects of claudin-9*^wt^* and claudin-9*^F35L^* on transepithelial resistance, we grew the Tet-off MDCK clones on microporous filters, and turned the expression of transfected proteins “on” and “off” (using doxycycline withdrawal and supplementation, respectively). Expression of claudin-9*^wt^* doubled the transepithelial resistance of Tet-off MDCK monolayers compared to that in control (“off”) cultures (Figure 5E). In contrast, the expression of claudin-9*^F35L^* lowered the transepithelial resistance of the MDCK epithelium (Figure 5F). Thus, expression of claudin-9*^wt^* made the MDCK monolayer electrically tighter, whereas claudin-9*^F35L^* expression made the epithelium leakier.

### Elimination of claudin-9 ion barrier function by the F35L amino acid substitution

**Figure 6:**
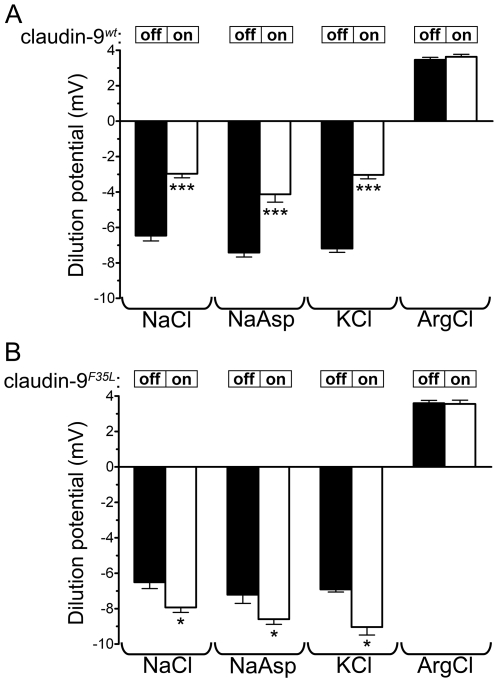
Claudin-9*^wt^* but not claudin-9*^F35L^* reduces the paracellular permeability for K^+^ and Na^+^. Dilution potential across (A) claudin-9*^wt^*- and (B) claudin-9*^F35L^*-transfected Tet-off MDCK cell clones, as assessed using Ussing chambers. Expression of the two claudin-9 constructs was turned “off” and “on” using doxycycline treatment as indicated. For baseline recordings, the basal and apical chambers were filled with the indicated buffers. For measurement of the dilution potential, the buffer on the basal side was replaced with a “½ buffer” that contained half the concentration of the main salt component indicated below each pair of columns (the osmolarity was maintained by substituting mannitol). Expression of claudin-9*^wt^* decreased the dilution potential in the NaCl, NaAsp, and KCl buffers, which suggests that this protein has a K^+^ and Na^+^ barrier function (mean±SEM, n = 3 in each of the 3 tested clones, paired *t* test, ***p<0.0001). Expression of claudin-9*^F35L^* did not affect the dilution potential in the ArgCl buffers, but increased the dilution potential in the NaCl, NaAsp, and KCl buffers (mean±SEM, n = 3 in each of the 3 tested clones, paired *t* test, *p<0.05).

The increased transepithelial resistance of claudin-9*^wt^*-expressing epithelia suggested that claudin-9*^wt^* reduced the paracellular permeability for some of the ions in the assay buffer. To characterize the ion permeability profile of both wild-type and mutant claudin-9, we performed dilution-potential assays in Ussing chambers, using the stable clones of claudin-9*^wt^* and claudin-9*^F35L^-*transfected Tet-off MDCK cells. In these experiments, the cells were seeded on Transwell filters, and the main salt component of the assay buffer was diluted 2-fold on one side of the MDCK monolayer, while osmolarity was maintained by adding mannitol. Asymmetric dilution of a salt on the apical versus basolateral side of an epithelial monolayer generates concentration gradients of the diluted anions and cations. This leads to transepithelial ion fluxes at different rates, depending on the anion and cation permeabilities of the epithelium. The unequal transepithelial fluxes of anions and cations generate a voltage termed the dilution potential. In control (i.e. claudin-9 “off”) MDCK monolayers, permeability to Na^+^ was greater than that to Cl^−^, as indicated by the apically negative dilution potential that was generated when both Na^+^ and Cl^−^ were diluted on the basolateral side. In MDCK monolayers expressing claudin-9*^wt^*, basolateral dilution of NaCl led to a much smaller voltage change (compare “on” and “off” bars in Figure 6A). This indicates that the expression of claudin-9*^wt^* either decreased the paracellular permeability to Na^+^ or increased the permeability to Cl^−^. To distinguish between these two possibilities, we replaced Cl^−^ with the poorly permeable anion aspartate (Asp). When the dilution potential assay was performed in the NaAsp buffer, claudin-9*^wt^* expression led to a reduction in the change in transepithelial voltage (Figure 6A). Thus, claudin-9*^wt^* decreased the paracellular permeability for Na^+^. In arginine chloride buffer (ArgCl), by contrast, claudin-9*^wt^* expression did not alter the dilution potential (Figure 6A), indicating that claudin-9*^wt^* did not affect the Cl^−^ permeability of MDCK cultures. Since the endolymph has a uniquely high K^+^ concentration amongst the extracellular fluids, we also tested the K^+^ permeability of claudin-9*^wt^* tight junctions. In KCl buffer, the dilution potential was reduced by the expression of claudin-9*^wt^* (Figure 6A). These results suggest that claudin-9*^wt^* formed a paracellular barrier to K^+^ and Na^+^, which is in agreement with the findings of a recent electrophysiological study in MDCK cells [39].

Next, we studied the ion permeability profile of MDCK epithelia in which claudin-9*^F35L^* expression was turned “on” or “off”. In contrast to the expression of wild-type protein, that of claudin-9*^F35L^* led to an increase in the dilution potential measured in the NaCl, NaAsp, and KCl buffers (compare “on” and “off” bars in Figure 6B). However, claudin-9*^F35L^* did not cause a change in the dilution potential when measured in the ArgCl buffer (Figure 6B). Thus, the mutant protein increased the K^+^ and Na^+^ permeability—but not the Cl^−^ permeability—of tight junctions in MDCK monolayers. Taken together, these data strongly suggest that claudin-9*^wt^* forms paracellular ion permeability barriers to Na^+^ and K^+^. Moreover, this cation barrier function is eliminated by the F35L substitution found in the claudin-9 protein of the nmf329 mouse strain.

### Claudin-9 expression and increased perilymphatic K^+^ concentration in the cochleas of nmf329 mice

**Figure 7:**
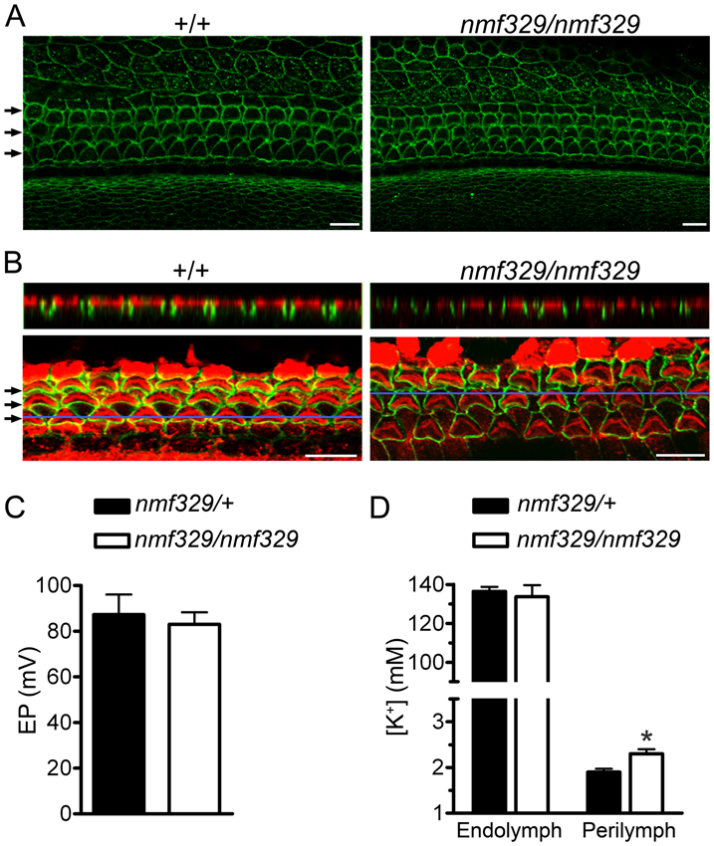
Claudin-9 expression and electrophysiological conditions in the cochleas of nmf329 mice. (A) Claudin-9 immunostaining of organ of Corti samples from wild-type and *nmf329/nmf329* mice (P5). OHC rows are indicated with arrows. Scale bars: 10 µm. (B) Claudin-9 immunostaining (green) of WGA-labeled (red) organ of Corti samples from wild-type and *nmf329/nmf329* mice (P5). Horizontal blue lines indicate the position of *Z* stack slices shown in the upper panels. OHC rows are indicated with arrows. Scale bars: 10 µm. (C) Endocochlear potential (EP) was measured in the cochleas of *nmf329/nmf329* mice and *nmf329/+* littermates *in vivo* (P70–P80). The EPs were not significantly different in the two groups (mean±SEM, n = 5 mice per group, unpaired *t* test). (D) *In vivo* measurement of K^+^ concentration in the endolymph and perilymph of *nmf329/nmf329* mice and *nmf329/+* littermates (P70–P80). The perilymphatic K^+^ concentration was higher in the *nmf329/nmf329* cochleas, whereas the endolymphatic K^+^ concentration was similar in cochleas of both genotypes (mean±SEM, n = 5 mice per group, unpaired *t* test, *p = 0.016).

To study the effect of the F35L substitution on claudin-9 localization *in vivo*, we performed immunohistochemistry experiments with a polyclonal antibody that recognizes the unique C-terminus of claudin-9. In the organ of Corti from both *nmf329/nmf329* mice and their wild-type littermates, staining was visible at the junctional complexes of hair cells and their supporting cells (Figure 7A). In addition to its expression in the organ of Corti, claudin-9 was also detected at the cell-cell borders in the inner sulcus, outer sulcus (Figure 7A), *stria vascularis*, and Reissner's membrane (data not shown), in both homozygous mutant and wild-type mice. The specificity of the immunostaining was verified in two separate sets of negative control experiments, in which we either replaced the anti-claudin-9 antibody with normal goat IgG or pre-incubated the anti-claudin-9 antibody with a blocking peptide (data not shown). Overall, our observations indicate that the F35L amino acid substitution did not prevent claudin-9 from localizing to the plasma membrane in the hearing organ.

Next, we investigated whether claudin-9*^F35L^* is localized to the apex of OHCs or dislocated towards the cell base. To visualize the apical surface of epithelial cells, we incubated cochlear preparations with fluorescently labeled wheat germ agglutinin (WGA) which binds to the specific carbohydrates on the surface of non-permeabilized cells. Following WGA labeling, tissue preparations were immunostained with the anti-claudin-9 antibody. Z-stack confocal microscopy images showed that both claudin-9*^wt^* and claudin-9*^F35L^* were localized to the apex of OHCs (Figure 7B; WGA and claudin-9 signals are shown individually in Figure S11). In addition, transmission electron microscopy images of freeze fracture replicas revealed that the tight junction strands in the organ of Corti of wild-type and *nmf329/nmf329* mice at P5 were similar (Figure S12). Furthermore, the expression pattern of another functionally important claudin in the cochlea—claudin-14 [40]—was not altered in the hearing organ of *nmf329/nmf329* mice (Figure S13). These results strongly suggest that the F35L alteration in claudin-9 does not affect the subcellular localization of claudin-9 and the organization of tight-junction strands in the OHCs.

To evaluate the electrophysiological consequences of the *claudin-9* mutation *in vivo*, we measured the endolymphatic and perilymphatic K^+^ concentrations, as well as the EP, in *nmf329/nmf329* mice and control (*nmf329/+*) littermates at P70–P80. These assays were performed in deeply anesthetized mice using double-barreled microelectrodes, as we have described for other mouse models [41],[42]. The EP and the endolymphatic K^+^ concentrations were similar in the *nmf329/nmf329* and *nmf329/+* mice, indicating that the *stria vascularis* functioned normally in the homozygous mutant animals (Figure 7C and 7D). However, the perilymphatic K^+^ concentration was significantly higher in the *nmf329/nmf329* mice than in their littermate controls (Figure 7D). These results show that the claudin-9 defect in the nmf329 line does not abolish the endocochlear K^+^ and voltage gradients.

### Lack of OHC degeneration in the claudin-9–defective cochleas under organ culture conditions

**Figure 8:**
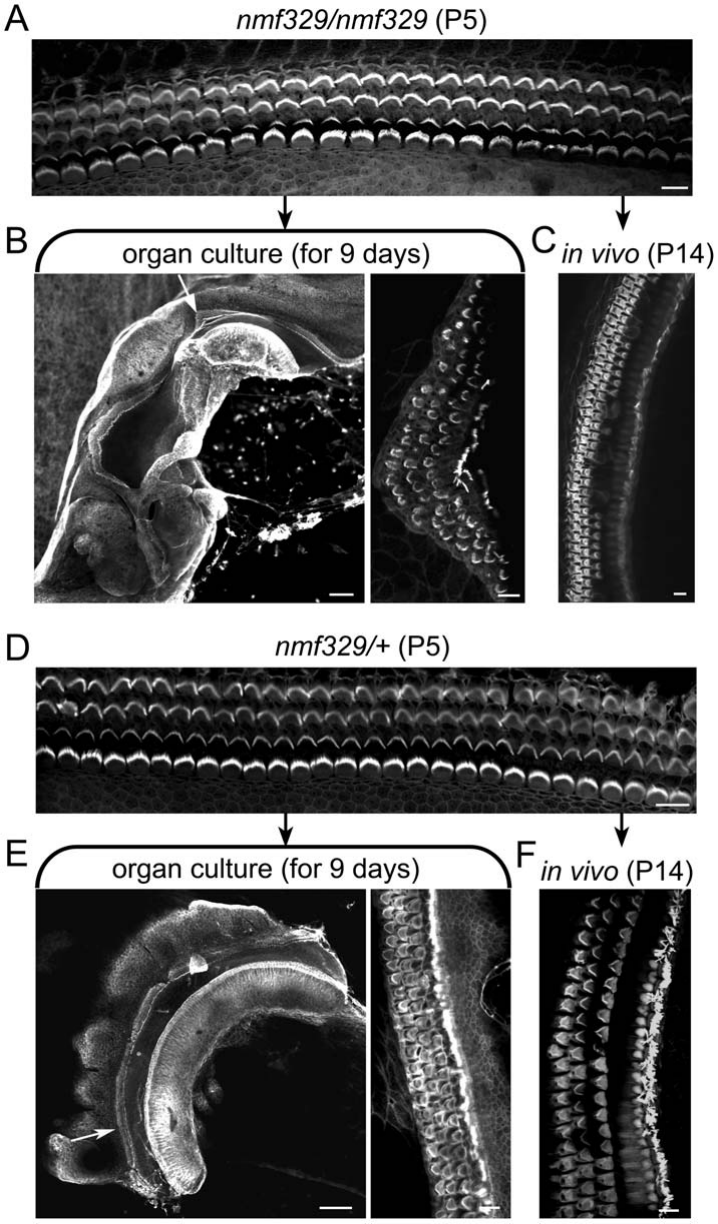
OHCs of nmf329 mice evade degeneration in organ culture. (A) Stereociliary bundles were visualized by F-actin staining in a whole-mount organ of Corti preparation from an *nmf329/nmf329* mouse at P5 (top panel). (B) F-actin staining of an organ of Corti sample that was explanted from an *nmf329/nmf329* mouse on P5 and maintained in organ culture for 9 days (n = 7). White arrow indicates the region shown at high magnification in the right panel. (C) A non-explanted cochlea from an *nmf329/nmf329* mouse (P14) was also analyzed for comparison. Each organ of Corti sample was dissected from the apical portion of the cochlea. The K^+^ concentration in the culture medium was 5.4 mM. (D) F-actin staining shows intact rows of OHCs in the cochlea of a control (*nmf329/+*) mouse at P5. (E) F-actin staining of an organ of Corti sample that was explanted from a control (*nmf329/+*) mouse on P5 and maintained in organ culture for 9 days (n = 8). Arrow indicates an area shown at higher magnification in the right panel. (F) A non-explanted cochlea from an *nmf329/+* mouse (P14) was stained with phalloidin-Alexa Fluor 488 for comparison. Scale bars in the low magnification panels are 100 µm (B and E), and 10 µm in all other panels.

We speculated that the small increase in the perilymphatic K^+^ concentration of *nmf329/nmf329* mice could reflect a spatially restricted defect of tight junctions. Also, the local K^+^ level close to the “leaky” epithelium may be higher than the K^+^ concentration in the bulk of the perilymph. To test whether extracellular conditions play a role in the hair-cell defect of *nmf329/nmf329* mice, we examined the rates of OHC degeneration in organ culture (5.4 mM K^+^ concentration). Organ of Corti samples from *nmf329/nm329* mice and control (*nmf329/+*) littermates were explanted on P5 (Figure 8A and 8D). The explanted tissue was maintained under previously described culture conditions that do not delay the differentiation of OHCs [43]. After 9 days of culturing, stereocilia were visualized by staining for F-actin. For comparison, organ of Corti samples were removed from 14-day old *nmf329/nmf329* mice, and were stained for F-actin. In contrast to the *in vivo* loss of OHCs, all three rows of OHCs survived when the organ of Corti samples from *nmf329/nmf329* mice were cultured *ex vivo* (compare Figure 8B and 8C). The OHCs of control (*nmf329/+*) littermates did not exhibit signs of degeneration either in organ culture or *in vivo* (Figure 8E and 8F). Thus, although the organs of Corti of *nmf329/nmf329* mice and littermate controls show different morphology *in vivo*, they are indistinguishable when maintained *ex vivo* (see OHC counts in Figure S14). These data are consistent with the notion that the degeneration of OHCs in the nmf329 mouse line is due to unfavorable extracellular conditions.

### Deletion of the *pou3f4* gene prevents the loss of OHCs in nmf329 mice

**Figure 9:**
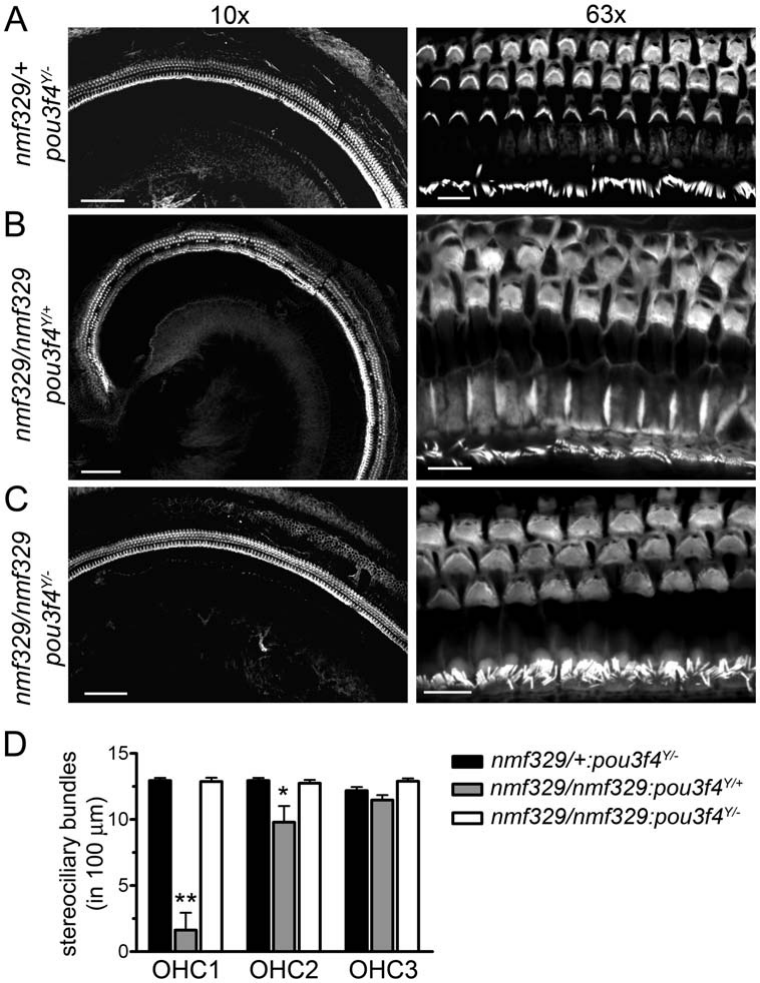
OHCs of nmf329 mice are rescued from degeneration by the deletion of *pou3f4*. (A–C) F-actin-based visualization of stereociliary bundles in the cochleas of (A) pou3f4-deficient (*nmf329/+:pou3f4^Y/−^*), (B) *nmf329* homozygous (*nmf329/nmf329:pou3f4^Y/+^*), and (C) *nmf329/pou3f4* double-mutant (*nmf329/nmf329:pou3f4^Y/−^*) mice at P14–15. Left panels are low magnification (10x objective) images for general overview; scale bars: 100 µm. Right panels show high magnification (63× objective) images; scale bars: 10 µm. (D) Counts of ciliated OHCs in the mid-turn cochleas of pou3f4-deficient (*nmf329/+:pou3f4^Y/−^*), *nmf329* homozygous (*nmf329/nmf329:pou3f4^Y/+^*), and *nmf329/pou3f4* double-mutant (*nmf329/nmf329:pou3f4^Y/−^*) mice at P14–15. OHC1, OHC2 and OHC3 indicate the first, second, and third row of OHCs, respectively. Data are mean±SEM (n = 6–7; one-way ANOVA, p<0.0001 for OHC1, p = 0.018 for OHC2; p = 0.16 for OHC3; *post hoc* Dunnett's test, control group is *nmf329/+:pou3f4^Y/−^*, **p<0.01, *p<0.05).

The pou3f4 transcription factor is required for the generation of EP, but it is not necessary for the development of hair cells [44]. In the nmf329 line, the loss of OHCs occurs after the endocochlear K^+^ concentration gradient is established (P2–P8 [45]) but coincides with the development of EP (P8–P16 [46]). Therefore, we tested whether the EP-dependent increase in the K^+^-driving force contributes to hair-cell loss in the *claudin-9* mutant mice *in vivo*. EP in the claudin-9-defective cochleas was reduced by breeding *nmf329* homozygotes with a mouse line lacking the *pou3f4* gene. Animals in the second backcross generation were examined for potential alterations in the morphology of OHC at P14–15. Actin staining of organ of Corti samples indicated that OHCs were intact in the *nmf329/+*:*pou3f4^Y/−^* animals (Figure 9A), which is consistent with previous histological analyses of *pou3f4^Y/−^* mice [44],[47]. *Nmf329/nmf329* mice with an unaltered *pou3f4* gene (*nmf329/nmf329*:*pou3f4^Y/+^*) showed widespread loss of OHCs at P14 (Figure 9B). In contrast, *nmf329/nmf329* littermates in which the *pou3f4* gene was deleted (*nmf329/nmf329:pou3f4^Y/−^*) exhibited a morphologically intact organ of Corti with three rows of OHCs (Figure 9C and 9D). Thus, deletion of the *pou3f4* gene in the nmf329 line prevented the loss of OHCs.

## Discussion

In this study, we show that claudin-9 deficiency is associated with deafness. We also show that wild-type claudin-9 is a paracellular ion permeability barrier for Na^+^ and K^+^, and that the *claudin-9* mutation of deaf nmf329 mice eliminates the ion barrier function of the encoded protein without preventing its plasma membrane localization. In addition to being expressed in the cochlea, claudin-9 has been detected in the vestibular system [35], liver [48], and developing kidney [49], yet *claudin-9* mutant mice exhibited no signs of vestibular, hepatic, or renal defects. Thus, the ion barrier function of claudin-9 is essential in the cochlea, but appears to be dispensable in other organs.

Sound detection requires uniquely large K^+^ and voltage gradients across the sensory hair cells of the hearing organ. During sound stimulation, hair cells are depolarized by an apical influx of K^+^ ions. Subsequently, hair cells repolarize as K^+^ ions are exported into the basolateral fluid. An increase in the K^+^ concentration in the basolateral fluid is known to inhibit repolarization and to be toxic to hair cells [30],[32]. Therefore, the basolateral fluid of hair cells must be insulated from the K^+^-rich apical milieu. This insulation is likely to be impaired in the nmf329 mice because: 1) the *nmf329* mutation eliminated the K^+^ barrier function of claudin-9, 2) the timing of hair-cell degeneration in the nmf329 mice coincided with the development of EP, 3) the OHCs of *claudin-9* mutant mice did not degenerate *ex vivo* under normal culture conditions, and 4) the OHCs of *claudin-9* mutant mice were rescued from degeneration by a second gene defect that reduces the K^+^-driving force in the cochlea. Since K^+^ can freely diffuse from the basolateral fluid of the hair cells to the perilymph in the *scala tympani*, it may seem surprising that the K^+^ concentration was only marginally elevated in the *scala tympani* of *claudin-9* mutant mice. However, the K^+^ concentration in the bulk of the perilymph may be significantly lower than the local K^+^ level in the basolateral fluid, because K^+^ is likely to become proportionally diluted as the distance from the “leaky” epithelium increases. In addition, simple diffusion is insufficient to protect the hair cells from K^+^ intoxication. Indeed, even during the normal hearing process, K^+^ is thought to be removed from the basolateral fluid by the K-Cl co-transporters of the supporting cells known as Kcc3 and Kcc4, rather than by diffusion, and genetic deletion of either *Kcc3* or *Kcc4* causes hair-cell degeneration [50],[51]. Thus, we suggest that the claudin-9 defect of nmf329 mice leads to toxic levels of K^+^ in the basolateral fluid of hair cells, but that K^+^ ions are diluted to a nearly physiological concentration as they diffuse across the basilar membrane into the *scala tympani*.

The extent of hair-cell degeneration in the nmf329 cochlea was not uniform throughout the organ. More OHCs were missing at the basal turn than at the apex, and the OHCs were more affected than the IHCs. A similar pattern of hair-cell loss is often observed in damaged cochleas. However, in nmf329 mice, the pattern of OHC loss was not mirrored by the frequency distribution of the ABR thresholds: we detected uniformly elevated auditory thresholds whether the sound stimulated the base or the apex of the cochlea (Figure 1C). These data suggests that although the majority of OHCs at the apex of the claudin-9-deficient cochlea survive, they do not function properly. Thus, a “sublethal” functional defect of OHCs may be ubiquitous in the claudin-9-deficient cochlea. This functional defect may culminate in the loss of OHCs, depending on the susceptibility of individual OHCs to the local basolateral K^+^ concentration.

IHCs are the primary sensory cells of the auditory system, whereas the OHCs are eletromechanical amplifiers that improve hearing sensitivity by more than 40 dB. A previous study showed that the ABR threshold to pure tone sounds was increased by 40 dB when the OHCs (but not the IHCs) in parts of the cochlea were destroyed by kanamycin treatment [52]. Furthermore, the hearing threshold was elevated by 40–60 dB in mice deficient of prestin, a molecule key to eletromechanical amplification by OHCs [53]. In the nmf329 mouse line, the auditory threshold was increased by ∼60 dB at P28 (Figure 1C), and the IHCs were morphologically intact (Figure 3). Thus, the phenotype of nmf329 mice is compatible with a predominantly OHC defect, and the IHCs seem to retain at least some sensory function in the mutant mice up to P28.

Claudin-9 is widely expressed in the inner ear [35], so it is surprising that the *claudin-9* mutation does not lead to reductions in either the endolymphatic K^+^ concentration or the EP. However, 10 other claudins have been detected in the inner ear [34],[35]. Some of these proteins are present at low levels or can be detected only in certain areas of the cochlea. Nevertheless, the collective expression pattern of multiple claudins may restrict the paracellular leak of claudin-9-deficient tight junctions to a limited area such as the organ of Corti. A localized K^+^ leak may be compensated by increased K^+^ secretion from the *stria vascularis*, and this could stabilize both the EP and the endolymphatic K^+^ concentration.

Claudin-9*^F35L^* was localized to tight junctions in transfected MDCK cells and in cochlear epithelial cells. Furthermore, heterologous expression of claudin-9*^F35L^* reduced the tightness of tight junctions in MDCK cell cultures and increased the paracellular permeability to K^+^ and Na^+^. Based on these data, we propose that claudin-9*^F35L^* is incorporated into tight-junction strands, resulting in the disruption of the paracellular cation barrier. Thus, a phenotype might be expected not only in the *nmf329/nmf329* mice but also in the *nmf329/+* animals. However, the *nmf329/+* mice exhibited normal auditory thresholds and intact OHCs at each time point investigated. This suggests that tight-junction strands containing both claudin-9*^wt^* and claudin-9*^F35L^* are sufficiently tight to prevent cochlear degeneration. Alternatively, claudin-9*^wt^* and claudin-9*^F35L^* may be sorted into separate tight-junction strands, and the number of claudin-9*^wt^*-containing strands may be sufficient to maintain the paracellular cation barrier. An additional possibility is that claudin-9*^wt^* is expressed at a higher level than claudin-9*^F35L^* in the heterozygous mice.

The claudin-9 sequence is highly conserved between mouse and human (Figure S9), which raises the possibility that a claudin-9 deficiency may cause hearing loss not only in mice but also in humans. Although no deafness gene has been mapped to human chromosome 16p13 where *claudin-9* is located, a large number of deafness loci likely await recognition and mapping [54]. Interestingly, a DNA donor has already been identified with a nonsynonymous SNP (id#: rs34769999) in *claudin-9* that leads to a non-conservative amino acid substitution in the encoded protein (R116C). Although the DNA donor carrying this nonsynonymous SNP was heterozygous for the R116C alteration, future studies may identify deaf patients homozygous for genetic alterations in *claudin-9*.

In addition to claudin-9, several other claudins have been detected in the junctional complexes of the organ of Corti [34]. Most notably, claudin-14 has been localized to the junctional complexes of hair cells and supporting cells [22],[35]; mutations in *claudin-14* have been shown to cause extensive hair-cell loss and deafness [22],[40]. Thus, the phenotypes of *claudin-9* mutant mice and *claudin-14* “knock-out” animals are similar. In the junctional complexes of OHCs, claudin-14 and claudin-9 are sorted into two separate subdomains; claudin-14 is found only in the most apical tight-junction strands, whereas claudin-9 is detected solely in the deeper (subapical) strands [34]. In addition, both claudin-9 and claudin-14 form paracellular ion permeability barriers for K^+^ (Figure 6 and ref. [22]). Therefore, in the organ of Corti, claudin-14 would be expected to render claudin-9 redundant. In contrast to this expectation, analysis of the nmf329 mice suggests that not only the apical tight-junction strands, but also those that are subapical, can contribute significantly to the ion barrier capacity of junctional complexes. In summary, our characterization of the nmf329 mouse line not only reveals the biological significance of claudin-9, but also provides insight into the functional architecture of tight-junction complexes in the hearing organ.

## Materials and Methods

### Mouse lines and genotyping

Heterozygous nmf329 mice (C57BL/6J-nmf329/J) and hemizygous *pou3f4* mutant mice (C3HeB/FeJ-Pou3f4del-J/J) were obtained from The Jackson Laboratory. Experimental procedures were approved by the Animal Care and Use Committees of the University of Iowa and Kansas State University. For genotyping, the one-nucleotide difference between the wild-type and mutant *claudin-9* alleles was detected by PCR using HotStart Taq DNA polymerase (Qiagen), tail DNA extracts, a forward primer that anneals to a sequence identical in the wild-type and mutant genes (5′-TGGTTCATGGCAGATCTGGAGG-3′), and HPLC-purified reverse primers whose 3′ ends anneal to the affected nucleotide (5′-ACGATGCTGTTGCCGATG/A-3′ for the wild-type and *nmf329* alleles, respectively). The presence of the *pou3f4* allele was detected by PCR using the following primers: 5′-CACTCTGATGAAGAGACTCCAAC-3′ and 5′-CACCGTGTGCGAATAAACCTC-3′.

### Mapping of the *nmf329* locus

The nmf329 mice were crossed to the A/J strain. Female F1 mice from the intraspecific cross were backcrossed to *nmf329/nmf329* animals. F2 progeny were generated and analyzed for both hearing threshold (ABR measurement) and crossovers (SNP detection). Scanning of the Mouse Genomic Informatics database revealed 9 suitable SNPs over an 8-megabase genomic region, in the following genes: *Vmn2r105* (rs29506797), *Zfp160* (rs33247972), *4930432O21Rik* (rs32299331), *Zfp758* (rs32470819), *Kctd5* (rs33185969), *Gbl* (rs33167092), *Tsc2* (rs33261332), *Lmf1* (rs29499590), and *Uhrf1bp1* (rs33716796). Short DNA sequences (∼500 bp) encompassing the SNPs were PCR amplified, and the PCR products were denatured by heat and re-annealed by slow cooling. Thus, if two different alleles were amplified, they formed heterodimers with a single nucleotide mismatch. For the detection of DNA heterodimers, the re-annealed PCR products were treated with a plant enzyme (CEL1) that cleaves DNA at points of mismatch [55]. DNA cleavage was analyzed by agarose gel electrophoresis.

### Cloning of claudin-9 and expression in Tet-off MDCK cells

Total RNA was isolated from the inner ear of wild-type and *nmf329/nmf329* mice and reverse transcribed. The coding regions of the claudin-9*^wt^* and claudin-9*^F35L^* cDNAs were PCR amplified and subcloned into the EYFP-C1 vector (Clontech). The EYFP-claudin-9*^wt^* and EYFP-claudin-9*^F35L^* fragments were further subcloned into the pUHD10–3 vector. Linearized plasmids were transfected into Tet-off MDCK II cells (Clontech) using the Effectene transfection reagent (Qiagen), and stable clones were isolated following hygromycin (300 µg/ml) selection. Expression of endogenous claudin-9 in MDCK cells were tested using RT-PCR, DNAse-treated MDCK cell RNA, and the following primers: 5′-CTTCAACAGCCCTTGAACTC-3′, and 5′-TAGTCCCTCTTGTCCAGCC-3′.

### Measurement of TER

EYFP-claudin-9*^wt^*- and EYFP-claudin-9*^F35L^*-transfected stable clones of Tet-off MDCK II cells were seeded onto Transwell inserts (9×10^4^ cells/cm^2^) and incubated in the presence or absence of doxycycline (100 ng/ml) for 8 days. At the end of the incubation period, the electrical resistance across the cultures was measured using a Millicell-ERS (Millipore) ohmmeter in the cell culture medium (DMEM). Electrical resistance was also measured across blank inserts. TER was calculated by subtracting the resistance measured across the blank inserts from the resistance measured across the inserts with the MDCK monolayers.

### Measurement of dilution potential

EYFP-claudin-9*^wt^*- and EYFP-claudin-9*^F35L^*-transfected stable clones of Tet-off MDCK II cells were seeded onto Transwell inserts (9×10^4^ cells/cm^2^) and incubated in medium with or without doxycycline (100 ng/ml) for 8 days. At the end of the incubation period, Transwells were mounted into a custom-made Ussing chamber system (Jim's Instruments, Iowa City, Iowa). The apical and basal chambers were filled with one of the following solutions: NaCl buffer, NaAsp buffer, ArgCl buffer, or KCl buffer. These four buffers differed only in their main salt component, as indicated by their names (140 mM NaCl, 140 mM NaAsp, 140 mM ArgCl, and 140 mM KCl); the other components were identical in every buffer (2 mM CaCl_2_, 1 mM MgCl_2_, 10 mM glucose, 5 mM Tris, pH was adjusted to 7.3 with HCl).

The Ussing chambers were connected to Ag/AgCl electrodes via 3 M KCl agar bridges, and the electrodes were connected to a Quick Data Acquisition DT9800 USB board (Data Translation, Inc, Marlborough, MA) to record the voltage between the apical and basal chambers. The asymmetry of voltage-sensing Ag/AgCl electrodes and the liquid junction potentials were corrected using an offset-removal circuit. During the experiment, buffers were maintained at 37°C and bubbled constantly with air. Dilution potentials were measured by replacing the buffer on the basal side with a “½ buffer”. In select experiments, the assay buffer was diluted on the apical side of MDCK cultures to verify that the absolute value of the dilution potential did not depend on the direction of the ion gradients. The ½ NaCl, ½ NaAsp, ½ ArgCl, and ½ KCl buffers contained half the concentration (70 mM) of the salt indicated by their names, and mannitol at a concentration of 140 mM (to maintain osmolarity); concentrations of the other buffer components (i.e. Ca^2+^, Mg^2+^, glucose, Tris, H^+^) were not altered. Buffer replacement was considered complete after 3 quick, successive washes with any given buffer.

### ABR measurements

ABR measurements were performed as previous described [56]. In brief, mice were anesthetized with ketamine and xylazine, and were placed on a heating pad in a sound-attenuating chamber. Needle electrodes were introduced just under the skin, with the active electrode placed between the ears above the vertex of the skull, the ground electrode between the eyes, and the reference electrode underneath the left ear. Mice were presented with 1024 pure-tone or click stimuli at a rate of 21.1/s (Intelligent Hearing Systems, Miami, FL). Responses were bandpass filtered (100–1500 Hz) and recorded for 12 ms. Thresholds were determined by increasing the sound intensity in 10 dB increments, followed by 5 dB increases and decreases to determine the lowest level at which a distinct ABR wave pattern could be recognized.

### Rotarod test

The balance and motor coordination of WT, *nmf329/+*, and *nmf329/nmf329* mice were studied using rotarod tests as previously described [57]. Mice were placed onto a cylinder rotating at fixed speed (10 rpm), and latency to fall was measured for a maximum of 180 s. Each mouse underwent 4 trials. The latency to fall was measured in the 2^nd^, 3^rd^, and 4^th^ trials.

### Histology and immunofluorescence

For routine histological examination, the temporal bones were fixed in 4% PFA for 24 hours (4°C) and decalcified in Immunocal solution (Decal Chemical Corporation). The heart, lung, ileum, colon, liver, and kidney were fixed in Prefer solution (Anatech Ltd). Samples were embedded in paraffin, sectioned, deparaffinized, and stained with hematoxylin and eosin. For F-actin labeling, WGA labeling and immunostaining, the organ of Corti was dissected from the cochlea and fixed in one of the following solutions: Prefer solution (WGA labeling, claudin-9 immunostaining and claudin-14 immunostaining), 4% PFA (F-actin labeling and CD68 immunostaining), 10% TCA (occludin immunostaining). MDCK cells were fixed in ethanol (30 min, 4°C) and acetone (3 min, −20°C). When visualization of the cell surface was required, WGA-Alexa Fluor 594 (10 µg/ml) (Invitrogen Corp.) was added to the specimen before permeabilization. Tissue samples were permeabilized with 0.5% Triton X-100 in PBS for 20 min, and blocked with BSA. The following reagents and antibodies were used: phalloidin-Alexa Fluor 488 (Invitrogen Corp.), goat anti-claudin-9 antibody (C-20, Santa Cruz Biotech Corp.), rabbit anti-claudin-14 antibody (Invitrogen Corp.), monoclonal rat anti-CD68-Alexa Fluor 488 antibody (AbD Serotec, Raleigh, NC, USA), monoclonal anti-occludin antibody labeled with either Alexa Fluor 488 or Alexa Fluor 594 (Invitrogen Corp.). The sensitivity of the anti-CD68 antibody was verified using activated peritoneal macrophages as previously described [58]. The specificity of the anti-claudin-9 antibody was verified by immunofluorescence in claudin-9– and vector–transfected HEK293 cells. The specificity of the immunostaining in the organ of Corti was tested by pre-incubating the anti-claudin-9 antibody with a blocking peptide (10 µg/µl, Santa Cruz Biotech Corp.) for 1 h before adding it to the tissue samples. In additional control experiments, anti-claudin-9 and anti-claudin-14 antibodies were replaced with normal IgG from goat and rabbit (Calbiochem), respectively. Secondary anti-goat and anti-rabbit antibodies were labeled with Alexa Fluor 488 (Invitrogen Corp.). Images were obtained using an LSM-510 confocal microscope (Carl Zeiss Inc.).

### Freeze-fracture analysis

Organ of Corti samples were fixed in 2.5% glutaraldehyde solution supplemented with 3 mM CaCl_2_ and buffered with cacodylate (0.1M, pH 7.2) for 2 hours at room temperature. Tissue samples were then cryoprotected in 30% glycerol, mounted on gold stubs with central wells, and frozen by plunging into liquid propane. After freezing, the samples were fractured in a Balzers 301 unit using a fracture knife, followed by platinum/carbon replication. Replicas were washed in household chlorine bleach, rinsed in distilled water, and retrieved on 200-mesh formvar coated copper grids. The images of the replicas were recorded using a JEOL JEM-1230 TEM microscope equipped with a Gatan Ultrascan 1000 camera.

### Measurements of EP and the K^+^ concentration *in vivo*

Adult mice (P70–P80) were anesthetized with 4% tribromoethanol (13 µl/g body weight i.p.), and the K^+^ concentration and the EP were measured using double-barreled microelectrodes, as described previously [41],[42]. The EP electrode was filled with 500 mM NaCl; the K^+^-selective barrel was silanized and the tip filled with a liquid ion exchanger (Fluka 60398, K^+^ ionophore I-Cocktail B) that was backfilled with 150 mM KCl. Measurements in the basal turn of the cochlea were made by a round-window approach, through the basilar membrane of the first turn. The K^+^-selective electrode was calibrated in solutions with known cation (K^+^ and Na^+^) concentrations *in situ* at 37°C.

### *Ex vivo* culture of the organ of Corti

Organ of Corti samples were dissected from the cochleas of *nmf329/nmf329* and *nmf329/+* mice on P5. The tissue pieces were mounted on poly-L-lysine-coated glass coverslips, and cultured for 9 days in Neurobasal-A medium containing N2 supplement, B-27 supplement, BDNF (50 ng/ml), and L-glutamine (0.5 mM) as previously described [43].

### Statistical analysis

Results are presented as the mean±SEM. The results were statistically evaluated using the paired *t* test, unpaired *t* test, or one-way ANOVA followed by a *post hoc* Dunnett's test, as indicated in the figure legends.

## Supporting Information

Figure S1 Early-onset hearing loss in the nmf329 strain. ABR thresholds (dB-SPL) to broadband click stimuli in wild-type (+/+), *nmf329*/+, and *nmf329/nmf329* mice at P16. Data are mean±SEM (n = 5; one-way ANOVA, p<0.0001; *post hoc* Dunnett's test, control group is +/+, ^**^p<0.01).
(0.42 MB TIF)

Figure S2 Normal balancing ability in the nmf329 strain. Time spent on fixed-speed rotating rod (10 rpm) before falling by wild-type (+/+), *nmf329*/+, and *nmf329/nmf329* mice (P28). The maximum duration of the test was 180 s (dotted horizontal line). Each mouse was subjected to 4 trials. The latency to fall was measured in the 2^nd^, 3^rd^, and 4^th^ trials. Data are mean±SEM (n = 4; 2-way ANOVA, p>0.05 for the genotype variable).
(1.03 MB TIF)

Figure S3 Normal overall structure of the cochlea in *nmf329/nmf329* mice. Actin staining of cochlear cryosections from wild-type (+/+) (A) and *nmf329/nmf329* (B) mice at P80. Red signal shows actin staining predominantly in the stria vascularis (SV) and the organ of Corti (OC). Numbers indicate cochlear half turns; RM, Reissner's membrane; SG, spiral ganglion. Scale bars: 400 µm.
(9.36 MB EPS)

Figure S4 Lack of inflammation in the organ of Corti of nmf329 mice. Immunostaining of organ of Corti samples from (A) an *nmf329/nmf329* mouse (P15) and (B) a control (+/+) littermate using an anti-CD68 antibody. (C) Primary culture of peritoneal macrophages stained with the anti-CD68 antibody (positive control). Left panels show the fluorescence signals; right panels show the corresponding bright-field images. All images were acquired using a 20× objective.
(4.81 MB TIF)

Figure S5 Low magnification images of F-actin-stained organ of Corti samples from nmf329 and wild-type mice. (A-F) Organ of Corti preparations from +/+ and *nmf329/nmf329* mice were stained with phalloidin-Alexa Fluor 488 to visualize the actin-rich structures including stereocilia. At P8, all three rows of OHCs are present in the cochlea of both +/+ (A) and *nmf329/nmf329* mice (B). At P14, the organ of Corti is undamaged in the +/+ mouse (C), whereas several stereociliary bundles of OHCs are missing from the nmf329 cochlea, especially from the first row (D). At P80, the control cochlea is intact (E), but many OHC bundles are missing from the *nmf329/nmf329* cochlea (F). Arrowheads indicate points where two images of the same cochlea were joined digitally to provide in-focus pictures for the entire tissue preparation. Scale bars: 100 µm.
(7.89 MB TIF)

Figure S6 Approximately 200 µm-long regions of F-actin stained organ of Corti samples from nmf329 and wild-type mice. The actin content of stereociliary bundles was visualized in the organ of Corti preparations from +/+ and *nmf329/nmf329* mice, using phalloidin-Alexa Fluor 488. At P8, the stereociliary bundles are present in all three rows of OHCs in the cochlea of +/+ (A) and *nmf329/nmf329* mice (B, C). At P14, the organ of Corti is intact in the +/+ mouse (D), whereas the basal (E) and apical turns (F) in the nmf329 cochlea lack numerous stereociliary bundles. At P80, the control cochlea is undamaged (G), but the *nmf329/nmf329* cochlea contains only a few OHCs at the basal turn (H, arrowheads), and most OHCs are missing from the first row at the apex (I). Panels E and F are epifluorescence images; all other panels are confocal microscopy images. Scale bars: 10 µm.
(5.76 MB TIF)

Figure S7 Counts of ciliated OHCs in the cochleas of nmf329 and wild-type mice at P8, P14, and P80. (A-F) F-actin-stained stereociliary bundles were counted in the first (OHC1), second (OHC2), and third (OHC3) rows of OHCs. Results are shown separately for the basal (A, C, and E) and apical (B, D, and F) portions of the cochlear samples at P8 (A and B), P14 (C and D) and P80 (E and F). Stereociliary bundles were counted in 300–500 µm long regions from 6–8 ears and normalized to 100 µm. Data are mean±SEM (unpaired t-test, ^*^p<0.05, ^**^p<0.01, ^***^p<0.001).
(0.58 MB TIF)

Figure S8 Phalangeal scars in the organ of Corti of nmf329 mice. Immunostaining of organ of Corti samples from heterozygous (A) and homozygous (B) nmf329 mice (P14) with an anti-occludin antibody. (A) In the heterozygous mouse, all three rows of OHCs are intact. Asterisks indicate one OHC in each row. (B) In the *nmf329/nmf329* mouse, large polygonal cells replace OHCs in the first row (gray arrow). In the second and third rows, a few OHCs are present (asterisks), whereas others are replaced by hexagonal and pentagonal cells in the reticular lamina. The rows of OHCs and IHCs are indicated by arrowheads and arrows, respectively. Scale bars: 10 µm.
(1.10 MB TIF)

Figure S9 Claudin-9 orthologs contain phenylalanine at position 35. Alignment of claudin-9 protein sequences from 8 different species. Black shading indicates residue identity; gray shading indicates aminoacyl group similarity. The arrow marks the phenylalanine residue of claudin-9 that is replaced with leucine in the nmf329 mice.
(2.01 MB EPS)

Figure S10 Localization of EYFP-claudin-9*^wt^*, EYFP-claudin-9*^F35L^*, and occludin in MDCK cells. MDCK cell clones expressing (A) EYFP-claudin-9*^wt^* (green) and (B) EYFP-claudin-9*^F35L^* (green) were immunostained with an anti-occludin antibody (lower panels, red signal) to visualize tight junctions. Scale bars: 10 µm.
(9.95 MB TIF)

Figure S11 Claudin-9 immunostaining of WGA-labeled organ of Corti samples from +/+ and *nmf329/nmf329* mice. The surface of organ of Corti samples from (A) wild-type and (B) *nmf329/nmf329* mice (P5) was labeled with WGA-Alexa Fluor 594 (upper panels) before immunostaining with an anti-claudin-9 antibody (lower panels). Scale bars: 10 µm.
(2.58 MB TIF)

Figure S12 Similar morphology of tight junction strands in the organ of Corti of wild-type and nmf329 mice. Freeze fracture replicas of apical junctions in the organ of Corti of wild-type (A) and nmf329 (B) mice at P5. Arrows indicate apical junctions between OHCs and Deiters' cells; arrowheads indicate junctional regions between two Deiters' cells. Scale bars: 0.5 µm.
(5.08 MB TIF)

Figure S13 Expression of claudin-14 in the organ of Corti of wild-type and nmf329 mice. Immunostaining of organ of Corti samples from (A) +/+ and (B) *nmf329/nmf329* mice with an anti-claudin-14 antibody at P5. Arrows indicate the OHC rows. Scale bars: 10 µm.
(1.65 MB TIF)

Figure S14 Counts of stereociliary bundles in cultured organ of Corti samples from heterozygous and homozygous nmf329 mice. Organ of Corti explants from *nmf329*/+ and *nmf329/nmf329* mice (P5) were cultured for 9 days, and stereociliary bundles were counted in the first (OHC1), second (OHC2), and third (OHC3) rows of OHCs. Counts are normalized to 100 µm. Data are mean±SEM (n = 7 and 8 in the groups; unpaired t-test, p>0.05).
(0.40 MB TIF)

## References

[1] Van Itallie CM, Anderson JM (2006). Claudins and epithelial paracellular transport.. Annu Rev Physiol.

[2] Furuse M, Tsukita S (2006). Claudins in occluding junctions of humans and flies.. Trends Cell Biol.

[3] Tsukita S, Furuse M (2002). Claudin-based barrier in simple and stratified cellular sheets.. Curr Opin Cell Biol.

[4] Shin K, Fogg VC, Margolis B (2006). Tight junctions and cell polarity.. Annu Rev Cell Dev Biol.

[5] Anderson JM, Van Itallie CM, Fanning AS (2004). Setting up a selective barrier at the apical junction complex.. Curr Opin Cell Biol.

[6] Van Itallie CM, Anderson JM (2004). The molecular physiology of tight junction pores.. Physiology (Bethesda).

[7] Colegio OR, Van Itallie C, Rahner C, Anderson JM (2003). Claudin extracellular domains determine paracellular charge selectivity and resistance but not tight junction fibril architecture.. Am J Physiol Cell Physiol.

[8] Amasheh S, Meiri N, Gitter AH, Schöneberg T, Mankertz J (2002). Claudin-2 expression induces cation-selective channels in tight junctions of epithelial cells.. J Cell Sci.

[9] Wen H, Watry DD, Marcondes MC, Fox HS (2004). Selective decrease in paracellular conductance of tight junctions: role of the first extracellular domain of claudin-5.. Mol Cell Biol.

[10] Alexandre MD, Jeansonne BG, Renegar RH, Tatum R, Chen YH (2007). The first extracellular domain of claudin-7 affects paracellular Cl^−^ permeability.. Biochem Biophys Res Commun.

[11] Mrsny RJ, Brown GT, Gerner-Smidt K, Buret AG, Meddings JB (2008). A key claudin extracellular loop domain is critical for epithelial barrier integrity.. Am J Pathol.

[12] Piontek J, Winkler L, Wolburg H, Müller SL, Zuleger N (2008). Formation of tight junction: determinants of homophilic interaction between classic claudins.. FASEB J.

[13] Umeda K, Ikenouchi J, Katahira-Tayama S, Furuse K, Sasaki H (2006). ZO-1 and ZO-2 independently determine where claudins are polymerized in tight-junction strand formation.. Cell.

[14] Krause G, Winkler L, Mueller SL, Haseloff RF, Piontek J (2008). Structure and function of claudins.. Biochim Biophys Acta.

[15] Itoh M, Furuse M, Morita K, Kubota K, Saitou M (1999). Direct binding of three tight junction-associated MAGUKs, ZO-1, ZO-2 and ZO-3, with the COOH termini of claudins.. J Cell Biol.

[16] Poliak S, Matlis S, Ullmer C, Scherer SS, Peles E (2002). Distinct claudins and associated PDZ proteins form different autotypic tight junctions in myelinating Schwann cells.. J Cell Biol.

[17] Hamazaki Y, Itoh M, Sasaki H, Furuse M, Tsukita S (2002). Multi-PDZ domain protein 1 (MUPP1) is concentrated at tight junctions through its possible interaction with claudin-1 and junctional adhesion molecule.. J Biol Chem.

[18] Hou J, Gomes AS, Paul DL, Goodenough DA (2006). Study of claudin function by RNA interference.. J Biol Chem.

[19] Angelow S, Schneeberger EE, Yu AS (2007). Claudin-8 expression in renal epithelial cells augments the paracellular barrier by replacing endogenous claudin-2.. J Membr Biol.

[20] Yu AS, Enck AH, Lencer WI, Schneeberger EE (2003). Claudin-8 expression in Madin-Darby canine kidney cells augments the paracellular barrier to cation permeation.. J Biol Chem.

[21] Van Itallie C, Rahner C, Anderson JM (2001). Regulated expression of claudin-4 decreases paracellular conductance through a selective decrease in sodium permeability.. J Clin Invest.

[22] Ben-Yosef T, Belyantseva IA, Hughes ED, Kawamoto K, Van Itallie CM (2003). Claudin 14 knockout mice, a model for autosomal recessive deafness DFNB29, are deaf due to cochlear hair cell degeneration.. Hum Mol Genet.

[23] Hou J, Paul DL, Goodenough DA (2005). Paracellin-1 and the modulation of ion selectivity of tight junctions.. J Cell Sci.

[24] Van Itallie CM, Rogan S, Yu A, Vidal LS, Holmes J (2006). Two splice variants of claudin-10 in the kidney create paracellular pores with different ion selectivities.. Am J Physiol Renal Physiol.

[25] Hou J, Renigunta A, Konrad M, Gomes AS, Schneeberger EE (2008). Claudin-16 and claudin-19 interact and form a cation-selective tight junction complex.. J Clin Invest.

[26] Alexandre MD, Lu Q, Chen YH (2005). Overexpression of claudin-7 decreases the paracellular Cl^−^ conductance and increases the paracellular Na^+^ conductance in LLC-PK1 cells.. J Cell Sci.

[27] Furuse M, Sasaki H, Tsukita S (1999). Manner of interaction of heterogeneous claudin species within and between tight junction strands.. J Cell Biol.

[28] Tsukita S, Furuse M, Ito M (2001). Multifunctional strands in tight junctions.. Nat Rev Mol Cell Biol.

[29] Salt AN, Jahn AF, Santos-Sacchi J, Jahn AF, Santos-Sacchi J (2001). Dynamics of the inner ear fluids.. Physiology of the Ear.

[30] Hibino H, Kurachi Y (2006). Molecular and physiological bases of the K^+^ circulation in the mammalian inner ear.. Physiology (Bethesda).

[31] Kitajiri S, Miyamoto T, Mineharu A, Sonoda N, Furuse K (2004). Compartmentalization established by claudin-11-based tight junctions in stria vascularis is required for hearing through generation of endocochlear potential.. J Cell Sci.

[32] Wangemann P (2006). Supporting sensory transduction: cochlear fluid homeostasis and the endocochlear potential.. J Physiol.

[33] Gow A, Davies C, Southwood CM, Frolenkov G, Chrustowski M (2004). Deafness in Claudin 11-null mice reveals the critical contribution of basal cell tight junctions to stria vascularis function.. J Neurosci.

[34] Nunes FD, Lopez LN, Lin HW, Davies C, Azevedo RB (2006). Distinct subdomain organization and molecular composition of a tight junction with adherens junction features.. J Cell Sci.

[35] Kitajiri SI, Furuse M, Morita K, Saishin-Kiuchi Y, Kido H (2004). Expression patterns of claudins, tight junction adhesion molecules, in the inner ear.. Hear Res.

[36] JAX Neuroscience Mutagenesis Facility (2004). Heritable mouse mutants from JAX NMF ENU Mutagenesis Program.. http://www.jax.org/nmf.

[37] Forge A (1985). Outer hair cell loss and supporting cell expansion following chronic gentamicin treatment.. Hear Res.

[38] Raphael Y, Altschuler RA (1991). Reorganization of cytoskeletal and junctional proteins during cochlear hair cell degeneration.. Cell Motil Cytoskeleton.

[39] Sas D, Hu M, Moe OW, Baum M (2008). Effect of claudins 6 and 9 on paracellular permeability in MDCK II cells.. Am J Physiol Regul Integr Comp Physiol.

[40] Wilcox ER, Burton QL, Naz S, Riazuddin S, Smith TN (2001). Mutations in the gene encoding tight junction claudin-14 cause autosomal recessive deafness DFNB29.. Cell.

[41] Wangemann P, Itza EM, Albrecht B, Wu T, Jabba SV (2004). Loss of KCNJ10 protein expression abolishes endocochlear potential and causes deafness in Pendred syndrome mouse model.. BMC Med.

[42] Wangemann P, Nakaya K, Wu T, Maganti RJ, Itza EM (2007). Loss of cochlear HCO3^-^ secretion causes deafness via endolymphatic acidification and inhibition of Ca^2+^ reabsorption in a Pendred syndrome mouse model.. Am J Physiol Renal Physiol.

[43] Fukuda J, Ishimine H, Tokunaga M (2004). Identification of live hair cells in rat cochlear sections in culture with FM1-43 fluorescent dye.. Neurosci Lett.

[44] Minowa O, Ikeda K, Sugitani Y, Oshima T, Nakai S (1999). Altered cochlear fibrocytes in a mouse model of DFN3 nonsyndromic deafness.. Science.

[45] Anniko M, Nordemar H (1980). Embryogenesis of the inner ear. IV. Post-natal maturation of the secretory epithelia of the inner ear in correlation with the elemental composition in the endolymphatic space.. Arch Otorhinolaryngol.

[46] Anniko M (1985). Histochemical, microchemical (microprobe) and organ culture approaches to the study of auditory development.. Acta Otolaryngol.

[47] Phippard D, Lu L, Lee D, Saunders JC, Crenshaw EB (1999). Targeted mutagenesis of the POU-domain gene Brn4/Pou3f4 causes developmental defects in the inner ear.. J Neurosci.

[48] Zheng A, Yuan F, Li Y, Zhu F, Hou P (2007). Claudin-6 and claudin-9 function as additional coreceptors for hepatitis C virus.. J Virol.

[49] Abuazza G, Becker A, Williams SS, Chakravarty S, Truong HT (2006). Claudins 6, 9, and 13 are developmentally expressed renal tight junction proteins.. Am J Physiol Renal Physiol.

[50] Boettger T, Hübner CA, Maier H, Rus tMB, Beck FX (2002). Deafness and renal tubular acidosis in mice lacking the K-Cl co-transporter Kcc4.. Nature.

[51] Boettger T, Rust MB, Maier H, Seidenbecher T, Schweizer M (2003). Loss of K-Cl co-transporter KCC3 causes deafness, neurodegeneration and reduced seizure threshold.. EMBO J.

[52] Ryan A, Dallos P (1975). Effect of absence of cochlear outer hair cells on behavioural auditory threshold.. Nature.

[53] Liberman MC, Gao J, He DZ, Wu X, Jia S (2002). Prestin is required for electromotility of the outer hair cell and for the cochlear amplifier.. Nature.

[54] Kochhar A, Hildebrand MS, Smith RJ (2007). Clinical aspects of hereditary hearing loss.. Genet Med.

[55] Oleykowski CA, Bronson Mullins CR, Godwin AK, Yeung AT (1998). Mutation detection using a novel plant endonuclease.. Nucleic Acids Res.

[56] Kiss PJ, Knisz J, Zhang Y, Baltrusaitis J, Sigmund CD (2006). Inactivation of NADPH oxidase organizer 1 results in severe imbalance.. Curr Biol.

[57] Nakano Y, Longo-Guess CM, Bergstrom DE, Nauseef WM, Jones SM (2008). Mutation of the Cyba gene encoding p22*^phox^* causes vestibular and immune defects in mice.. J Clin Invest.

[58] Ramprasad MP, Terpstra V, Kondratenko N, Quehenberger O, Steinberg D (1996). Cell surface expression of mouse macrosialin and human CD68 and their role as macrophage receptors for oxidized low density lipoprotein.. Proc Natl Acad Sci U S A.

