# Supplementary figures and images for: A Claudin-9–Based Ion Permeability Barrier Is Essential for Hearing
Source: PLoS Genet. 2009 Aug 21;5(8):e1000610. doi: 10.1371/journal.pgen.1000610 (PMC2720454; doi:10.1371/journal.pgen.1000610)

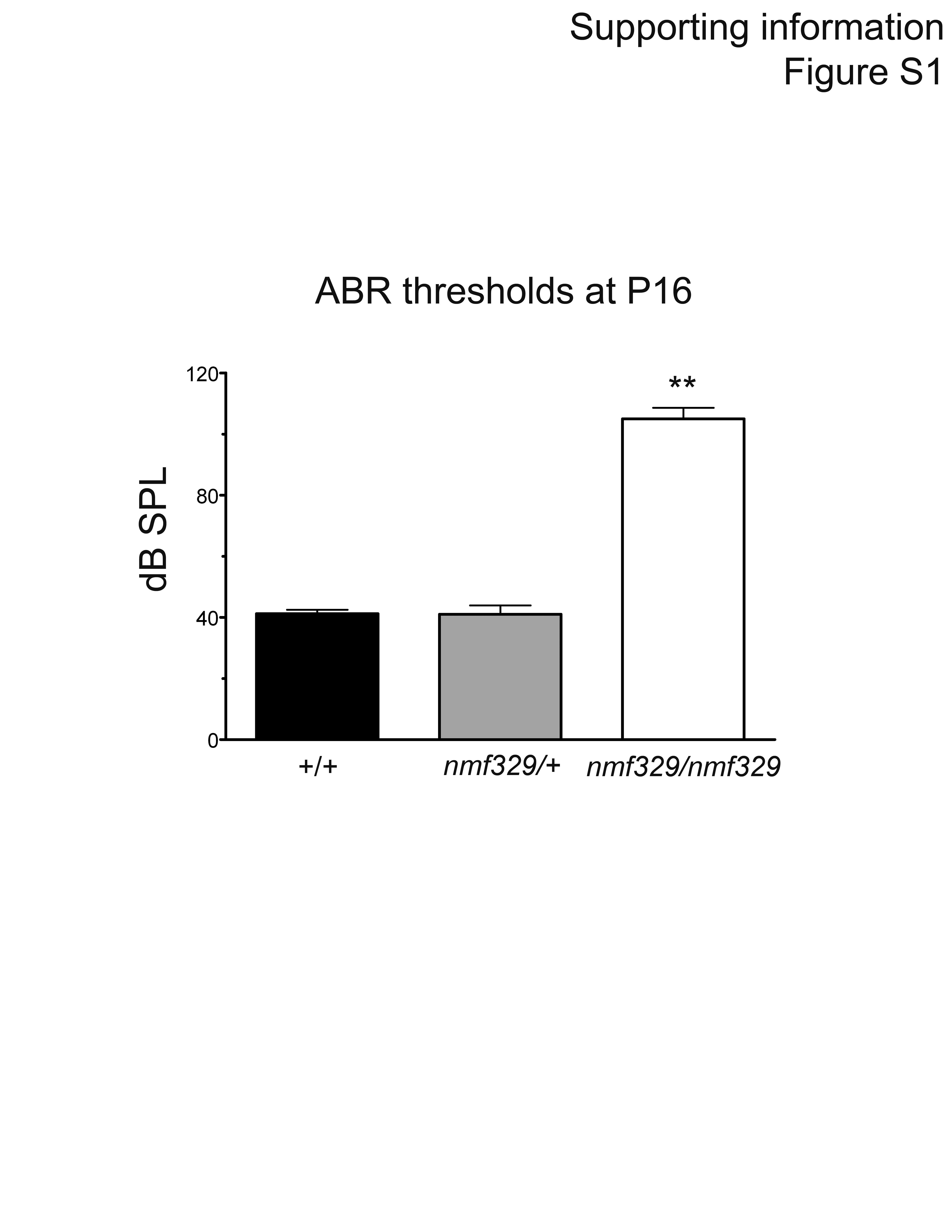

Supplement: Figure S1 — Early-onset hearing loss in the nmf329 strain. ABR thresholds (dB-SPL) to broadband click stimuli in wild-type (+/+), nmf329/+, and nmf329/nmf329 mice at P16. Data are mean±SEM (n = 5; one-way ANOVA, p<0.0001; post hoc Dunnett's test, control group is +/+, **p<0.01). (0.42 MB TIF) [file pgen.1000610.s001.tif]

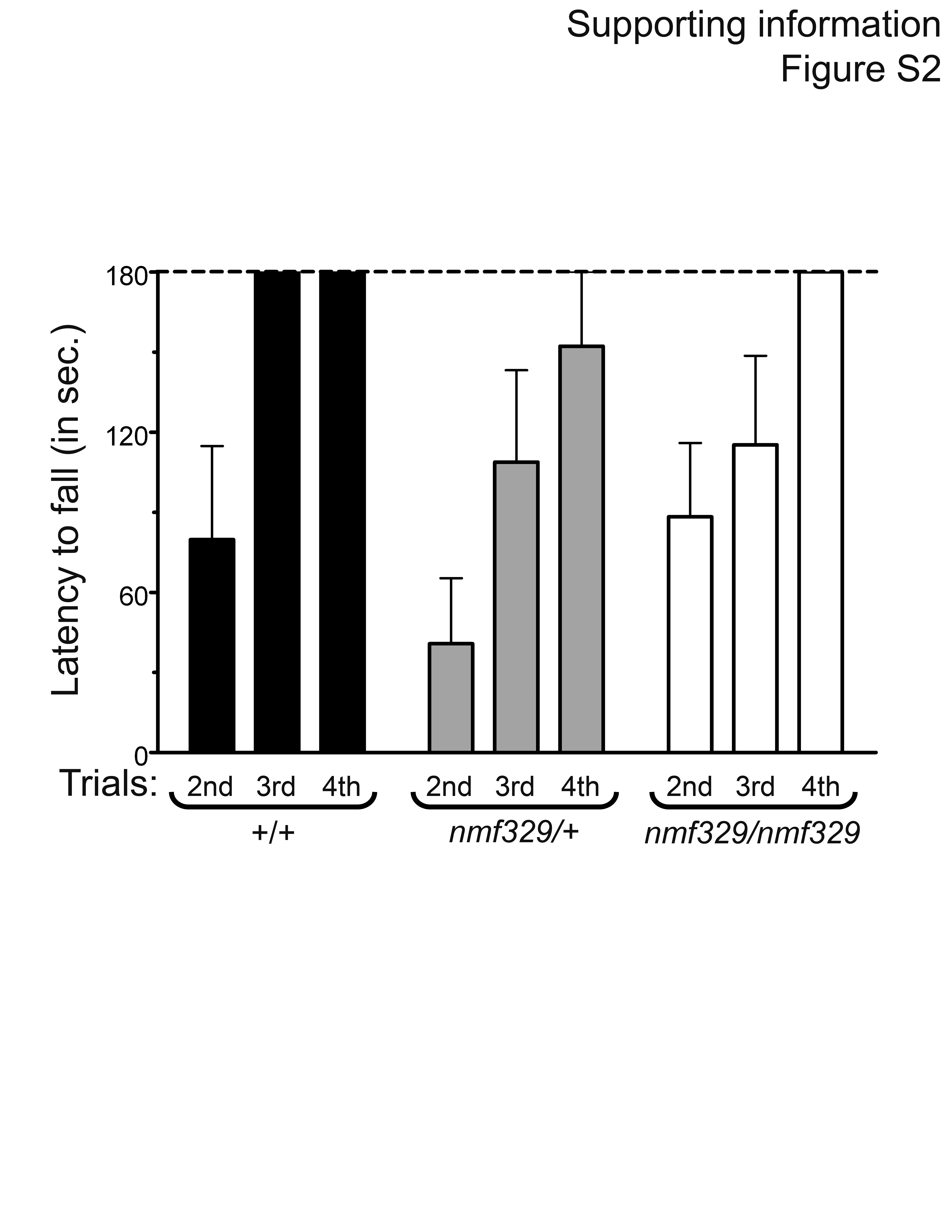

Supplement: Figure S2 — Normal balancing ability in the nmf329 strain. Time spent on fixed-speed rotating rod (10 rpm) before falling by wild-type (+/+), nmf329/+, and nmf329/nmf329 mice (P28). The maximum duration of the test was 180 s (dotted horizontal line). Each mouse was subjected to 4 trials. The latency to fall was measured in the 2nd, 3rd, and 4th trials. Data are mean±SEM (n = 4; 2-way ANOVA, p>0.05 for the genotype variable). (1.03 MB TIF) [file pgen.1000610.s002.tif]

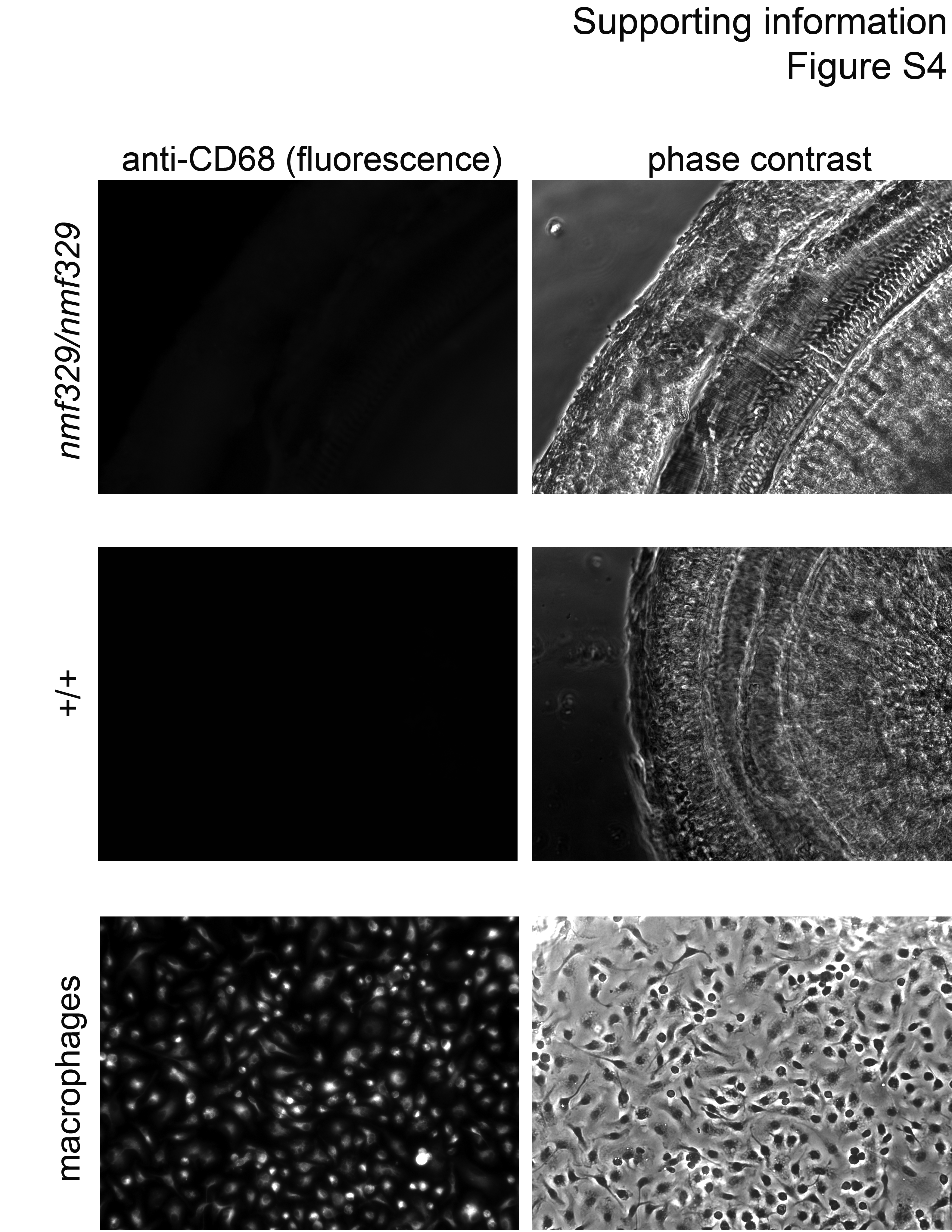

Supplement: Figure S4 — Lack of inflammation in the organ of Corti of nmf329 mice. Immunostaining of organ of Corti samples from (A) an nmf329/nmf329 mouse (P15) and (B) a control (+/+) littermate using an anti-CD68 antibody. (C) Primary culture of peritoneal macrophages stained with the anti-CD68 antibody (positive control). Left panels show the fluorescence signals; right panels show the corresponding bright-field images. All images were acquired using a 20× objective. (4.81 MB TIF) [file pgen.1000610.s004.tif]

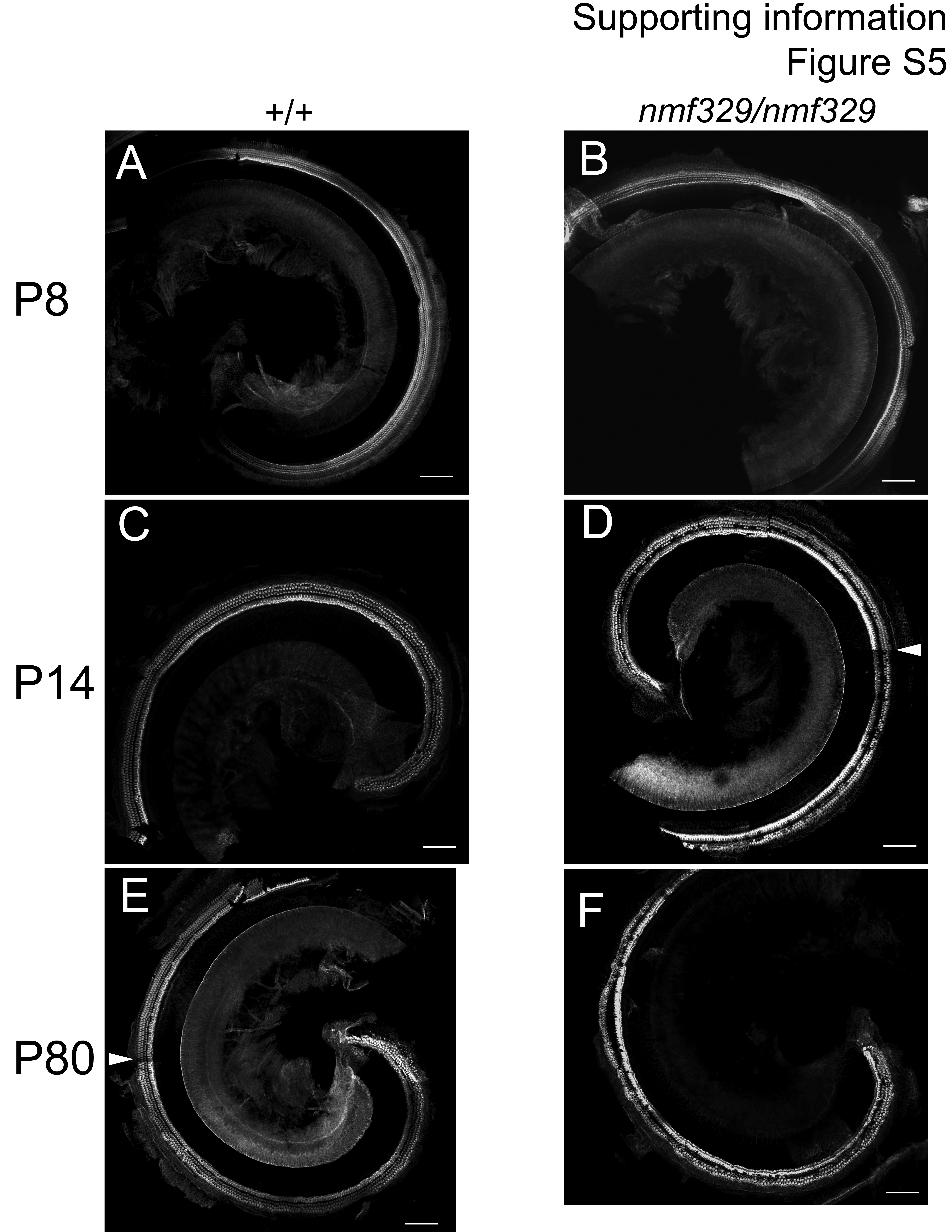

Supplement: Figure S5 — Low magnification images of F-actin-stained organ of Corti samples from nmf329 and wild-type mice. (A-F) Organ of Corti preparations from +/+ and nmf329/nmf329 mice were stained with phalloidin-Alexa Fluor 488 to visualize the actin-rich structures including stereocilia. At P8, all three rows of OHCs are present in the cochlea of both +/+ (A) and nmf329/nmf329 mice (B). At P14, the organ of Corti is undamaged in the +/+ mouse (C), whereas several stereociliary bundles of OHCs are missing from the nmf329 cochlea, especially from the first row (D). At P80, the control cochlea is intact (E), but many OHC bundles are missing from the nmf329/nmf329 cochlea (F). Arrowheads indicate points where two images of the same cochlea were joined digitally to provide in-focus pictures for the entire tissue preparation. Scale bars: 100 µm. (7.89 MB TIF) [file pgen.1000610.s005.tif]

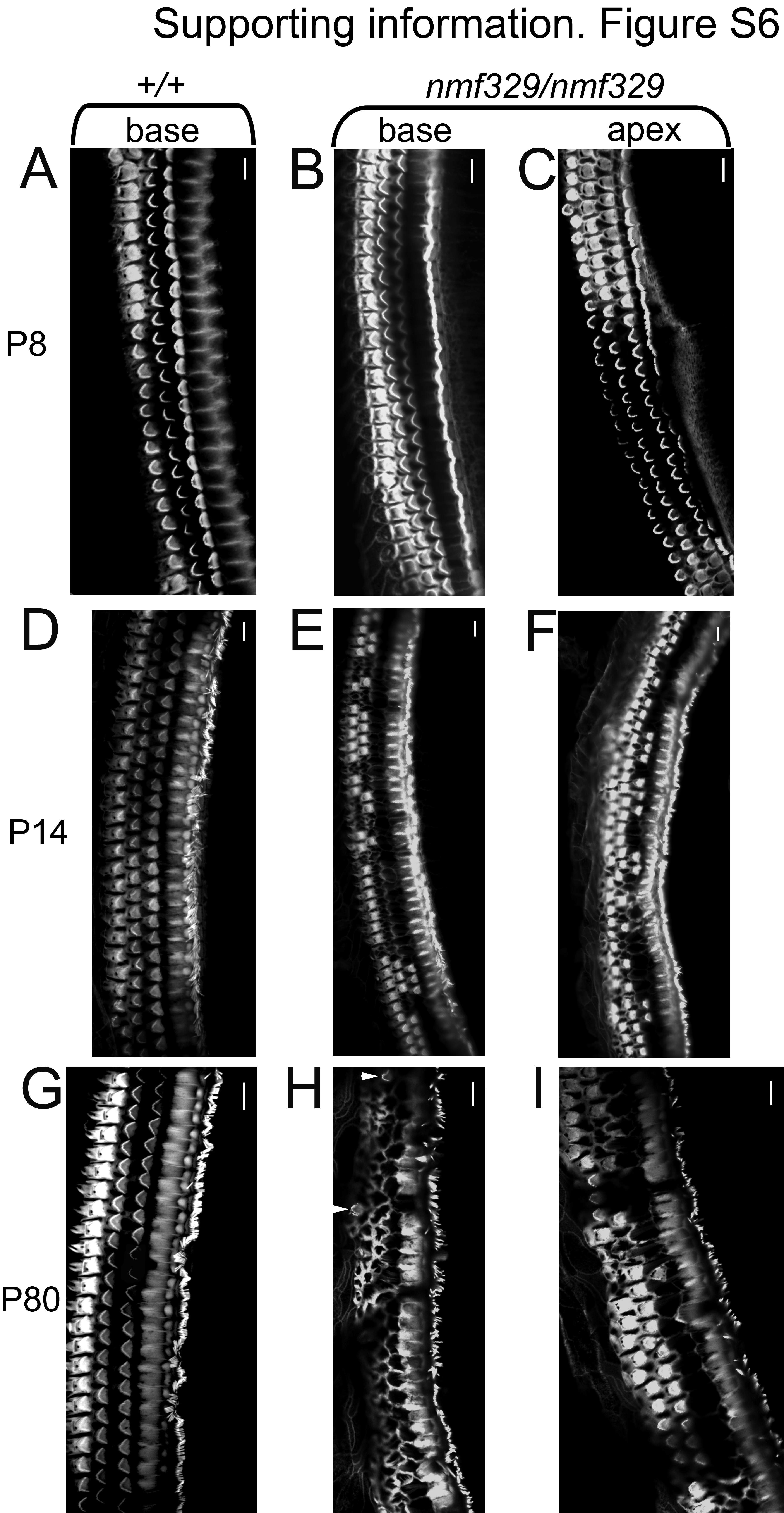

Supplement: Figure S6 — Approximately 200 µm-long regions of F-actin stained organ of Corti samples from nmf329 and wild-type mice. The actin content of stereociliary bundles was visualized in the organ of Corti preparations from +/+ and nmf329/nmf329 mice, using phalloidin-Alexa Fluor 488. At P8, the stereociliary bundles are present in all three rows of OHCs in the cochlea of +/+ (A) and nmf329/nmf329 mice (B, C). At P14, the organ of Corti is intact in the +/+ mouse (D), whereas the basal (E) and apical turns (F) in the nmf329 cochlea lack numerous stereociliary bundles. At P80, the control cochlea is undamaged (G), but the nmf329/nmf329 cochlea contains only a few OHCs at the basal turn (H, arrowheads), and most OHCs are missing from the first row at the apex (I). Panels E and F are epifluorescence images; all other panels are confocal microscopy images. Scale bars: 10 µm. (5.76 MB TIF) [file pgen.1000610.s006.tif]

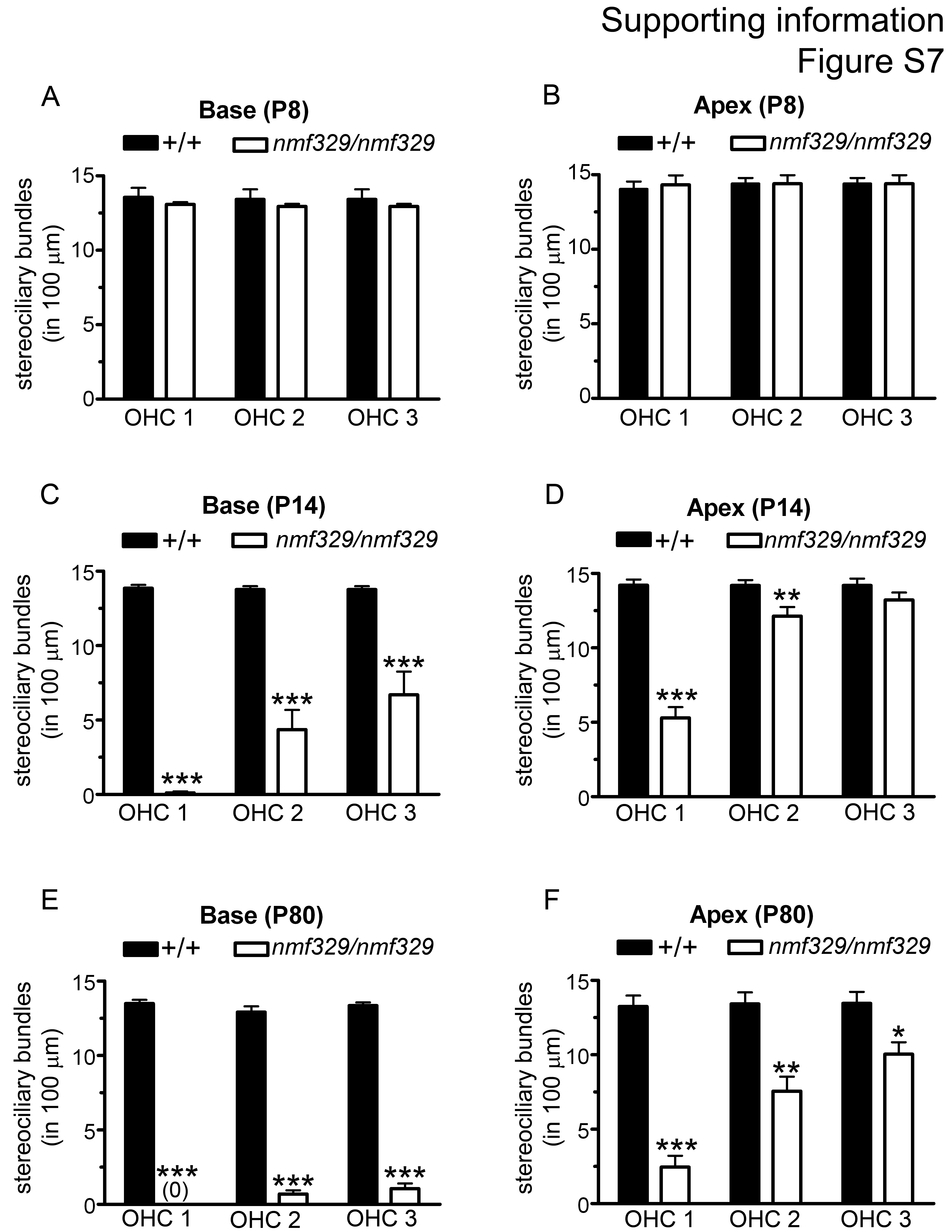

Supplement: Figure S7 — Counts of ciliated OHCs in the cochleas of nmf329 and wild-type mice at P8, P14, and P80. (A-F) F-actin-stained stereociliary bundles were counted in the first (OHC1), second (OHC2), and third (OHC3) rows of OHCs. Results are shown separately for the basal (A, C, and E) and apical (B, D, and F) portions of the cochlear samples at P8 (A and B), P14 (C and D) and P80 (E and F). Stereociliary bundles were counted in 300–500 µm long regions from 6–8 ears and normalized to 100 µm. Data are mean±SEM (unpaired t-test, *p<0.05, **p<0.01, ***p<0.001). (0.58 MB TIF) [file pgen.1000610.s007.tif]

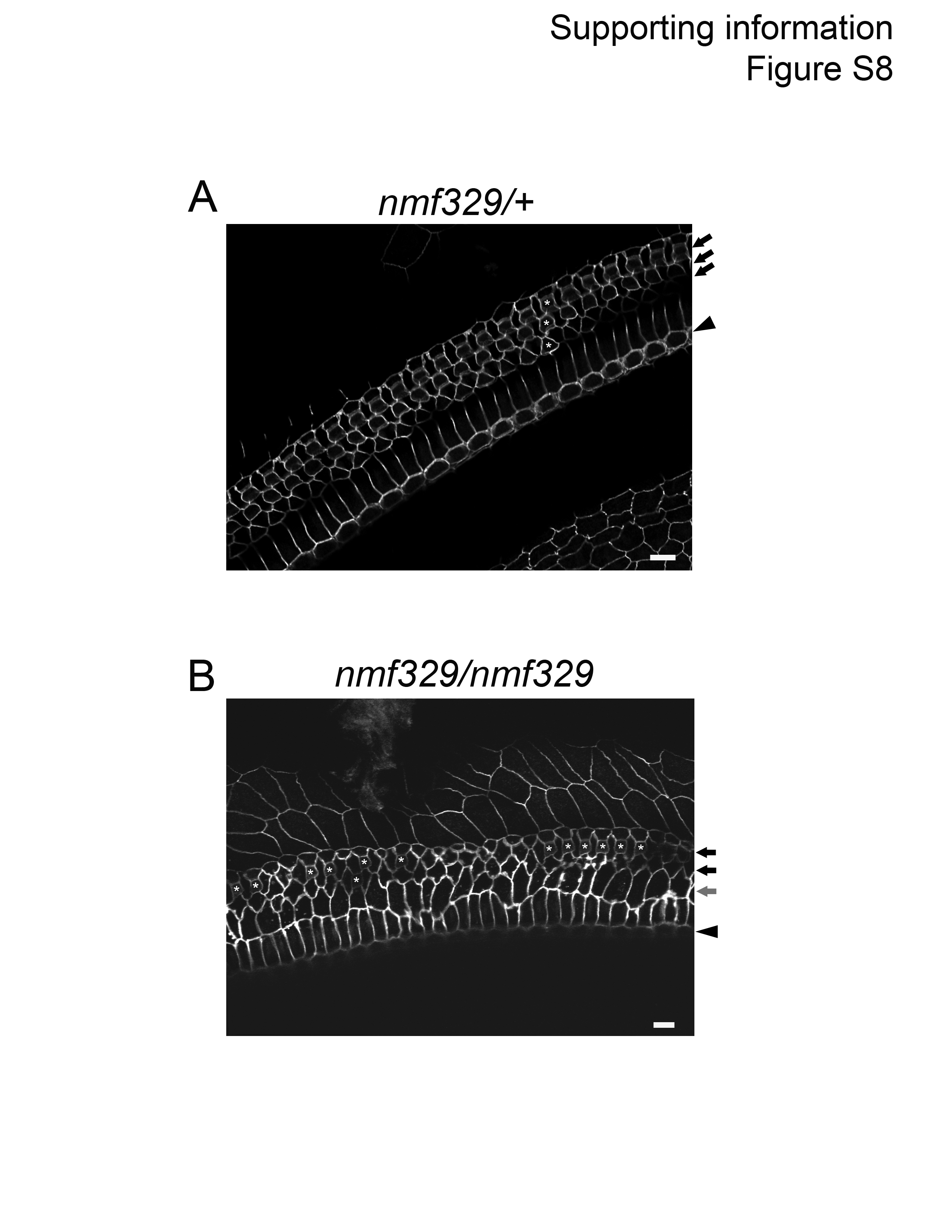

Supplement: Figure S8 — Phalangeal scars in the organ of Corti of nmf329 mice. Immunostaining of organ of Corti samples from heterozygous (A) and homozygous (B) nmf329 mice (P14) with an anti-occludin antibody. (A) In the heterozygous mouse, all three rows of OHCs are intact. Asterisks indicate one OHC in each row. (B) In the nmf329/nmf329 mouse, large polygonal cells replace OHCs in the first row (gray arrow). In the second and third rows, a few OHCs are present (asterisks), whereas others are replaced by hexagonal and pentagonal cells in the reticular lamina. The rows of OHCs and IHCs are indicated by arrowheads and arrows, respectively. Scale bars: 10 µm. (1.10 MB TIF) [file pgen.1000610.s008.tif]

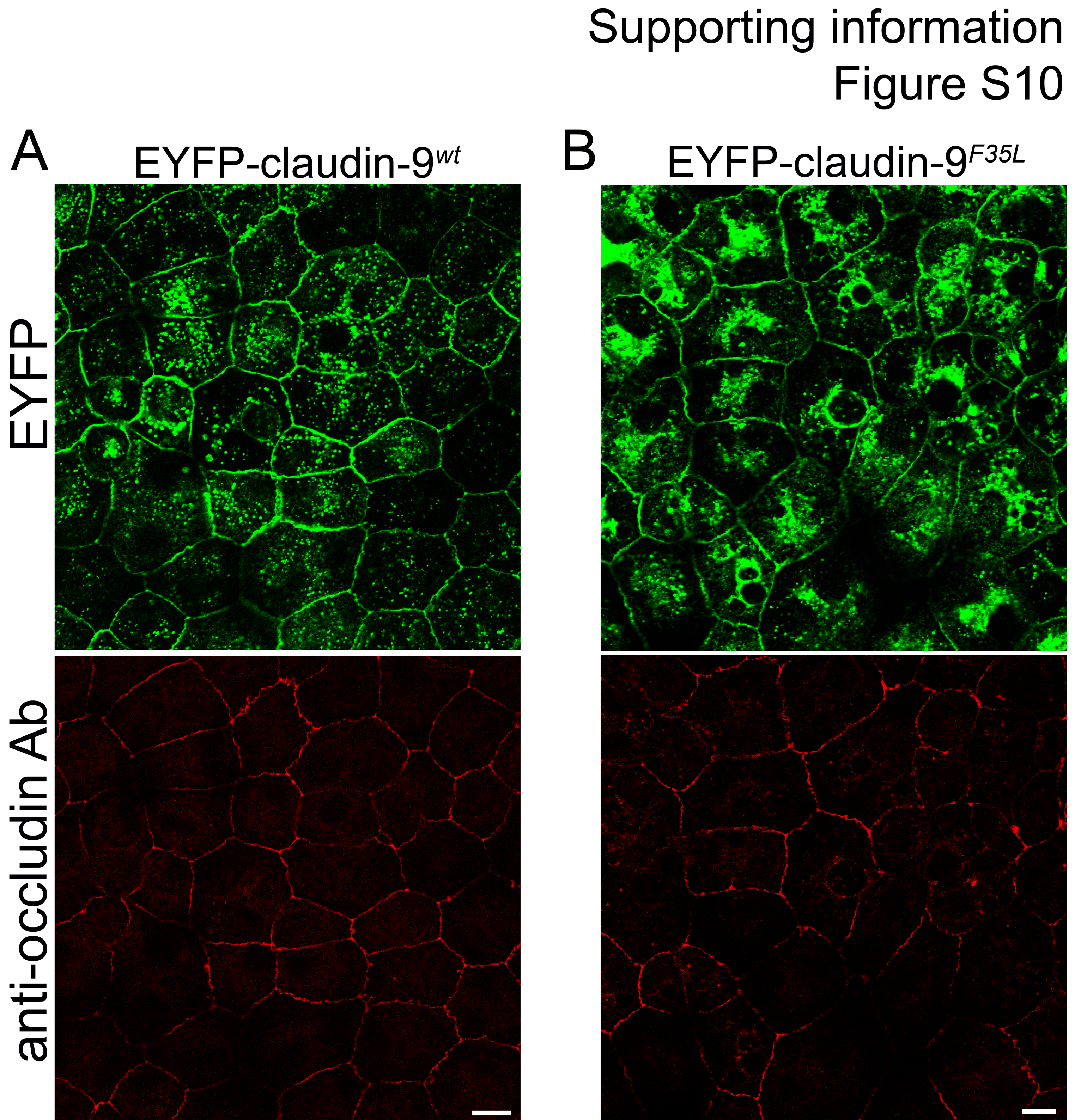

Supplement: Figure S10 — Localization of EYFP-claudin-9wt, EYFP-claudin-9F35L, and occludin in MDCK cells. MDCK cell clones expressing (A) EYFP-claudin-9wt (green) and (B) EYFP-claudin-9F35L (green) were immunostained with an anti-occludin antibody (lower panels, red signal) to visualize tight junctions. Scale bars: 10 µm. (9.95 MB TIF) [file pgen.1000610.s010.tif]

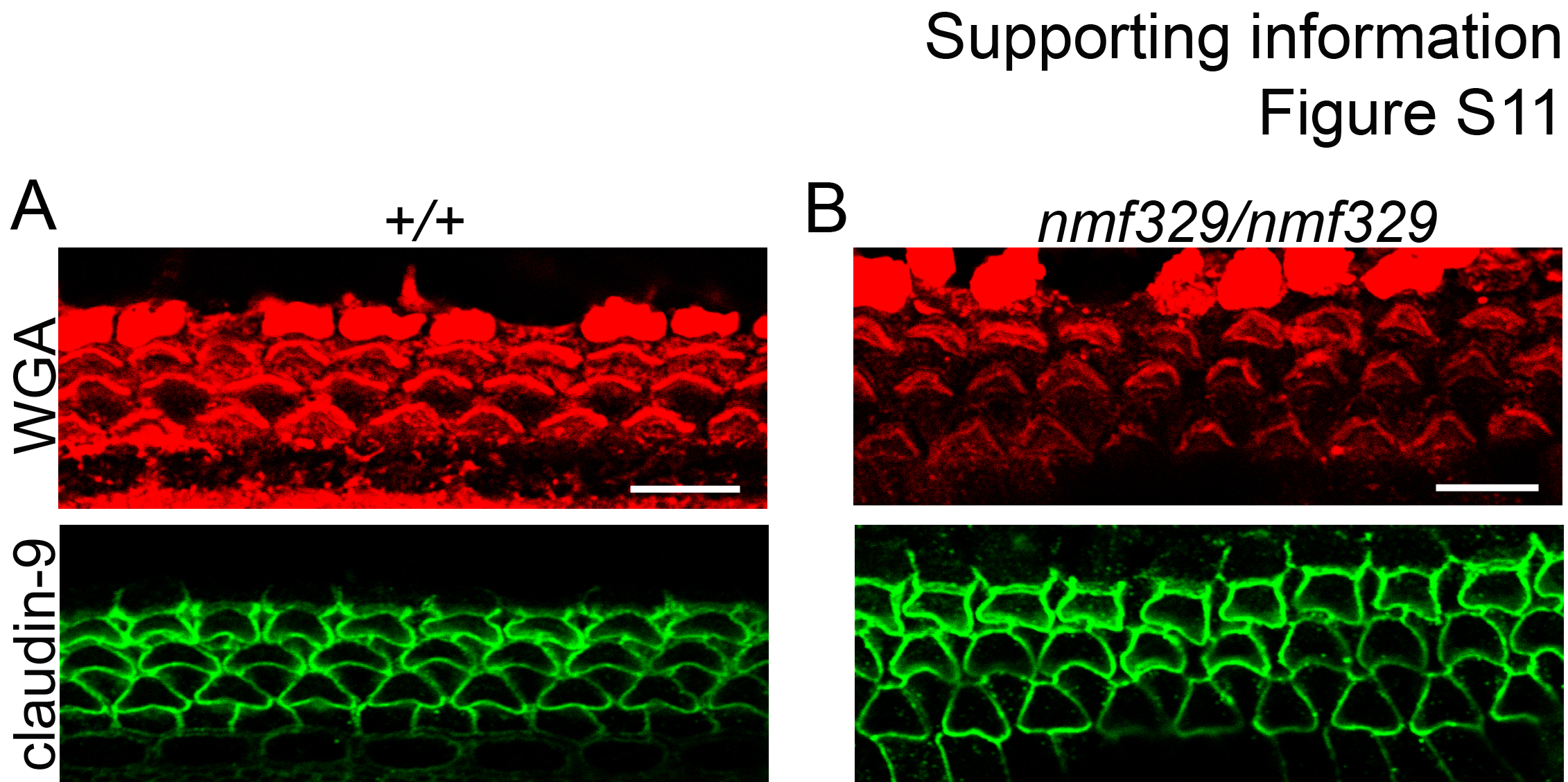

Supplement: Figure S11 — Claudin-9 immunostaining of WGA-labeled organ of Corti samples from +/+ and nmf329/nmf329 mice. The surface of organ of Corti samples from (A) wild-type and (B) nmf329/nmf329 mice (P5) was labeled with WGA-Alexa Fluor 594 (upper panels) before immunostaining with an anti-claudin-9 antibody (lower panels). Scale bars: 10 µm. (2.58 MB TIF) [file pgen.1000610.s011.tif]

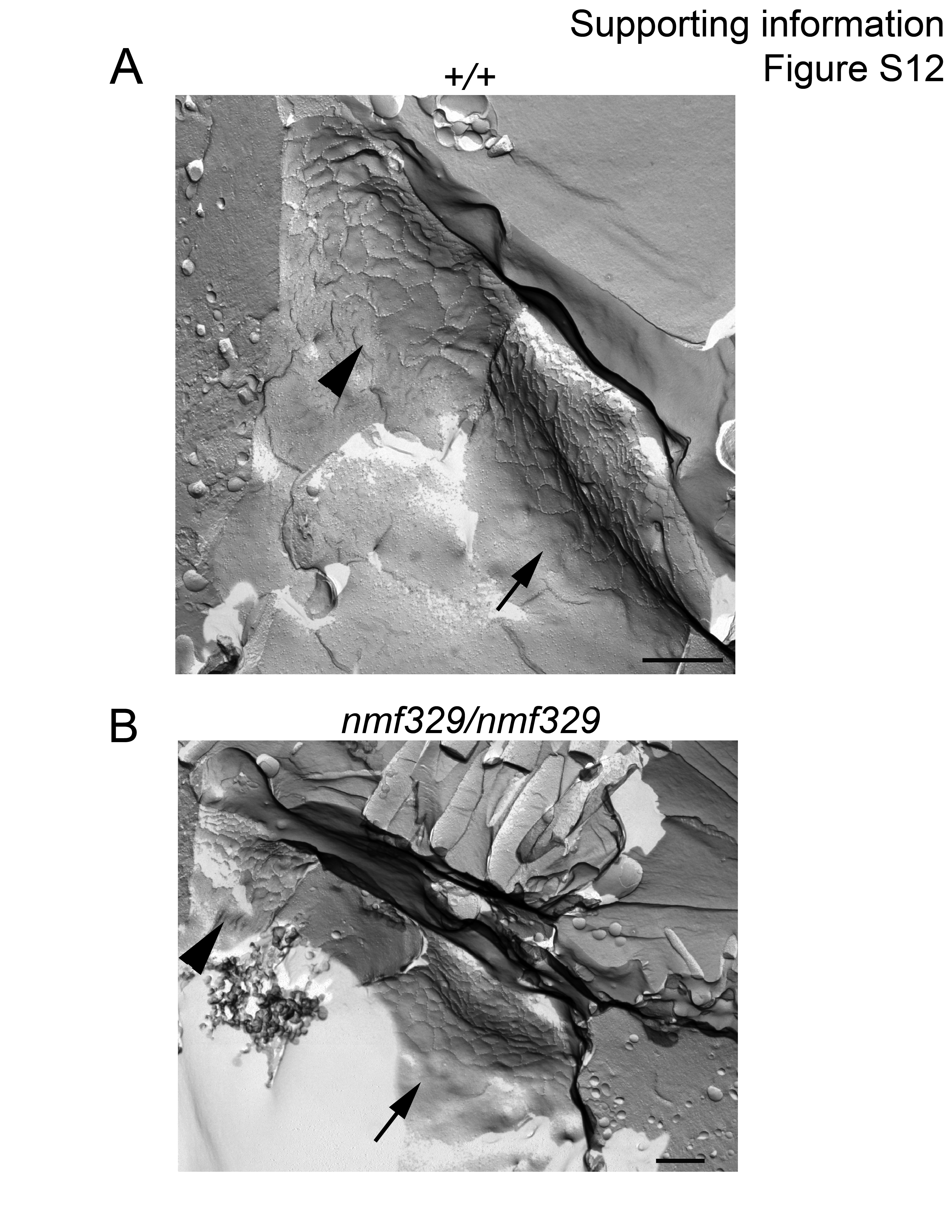

Supplement: Figure S12 — Similar morphology of tight junction strands in the organ of Corti of wild-type and nmf329 mice. Freeze fracture replicas of apical junctions in the organ of Corti of wild-type (A) and nmf329 (B) mice at P5. Arrows indicate apical junctions between OHCs and Deiters' cells; arrowheads indicate junctional regions between two Deiters' cells. Scale bars: 0.5 µm. (5.08 MB TIF) [file pgen.1000610.s012.tif]

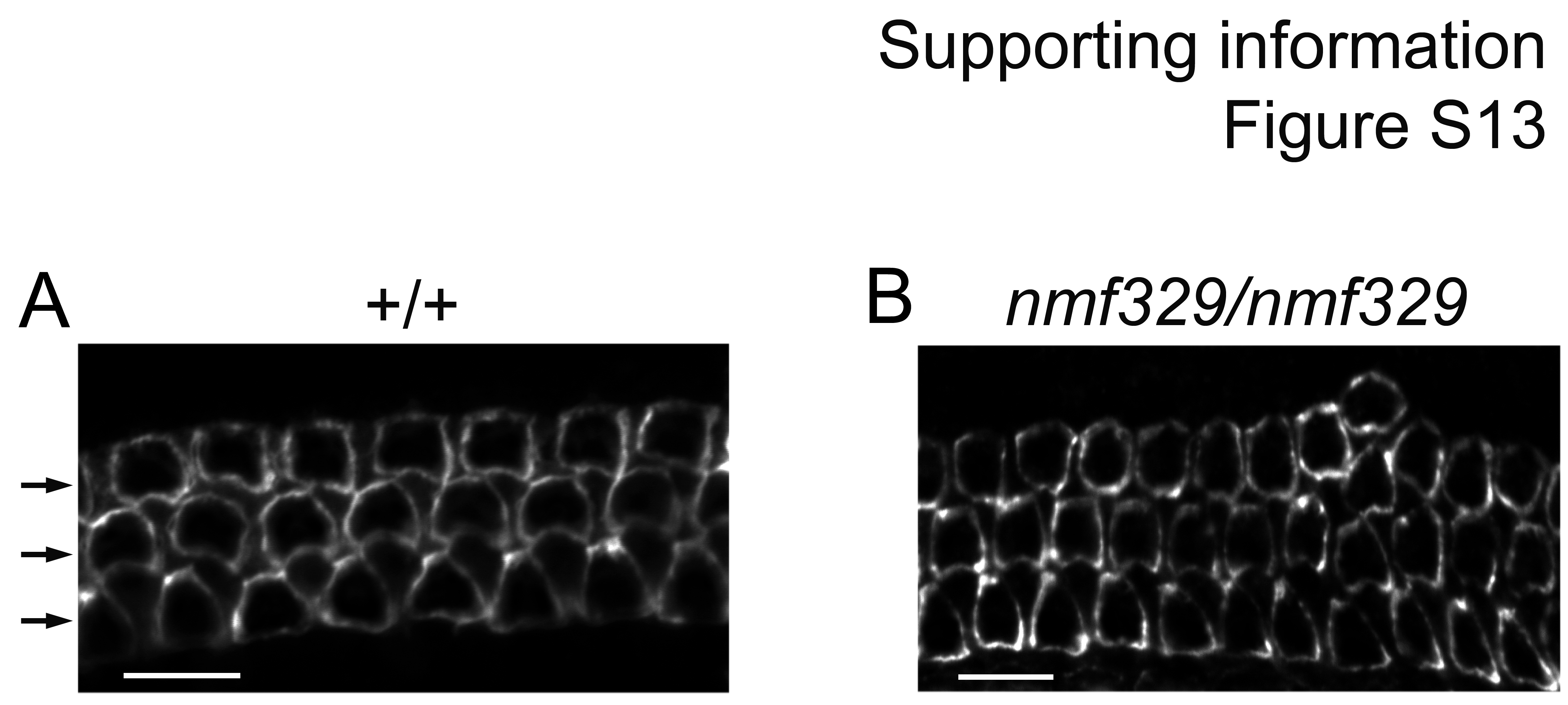

Supplement: Figure S13 — Expression of claudin-14 in the organ of Corti of wild-type and nmf329 mice. Immunostaining of organ of Corti samples from (A) +/+ and (B) nmf329/nmf329 mice with an anti-claudin-14 antibody at P5. Arrows indicate the OHC rows. Scale bars: 10 µm. (1.65 MB TIF) [file pgen.1000610.s013.tif]

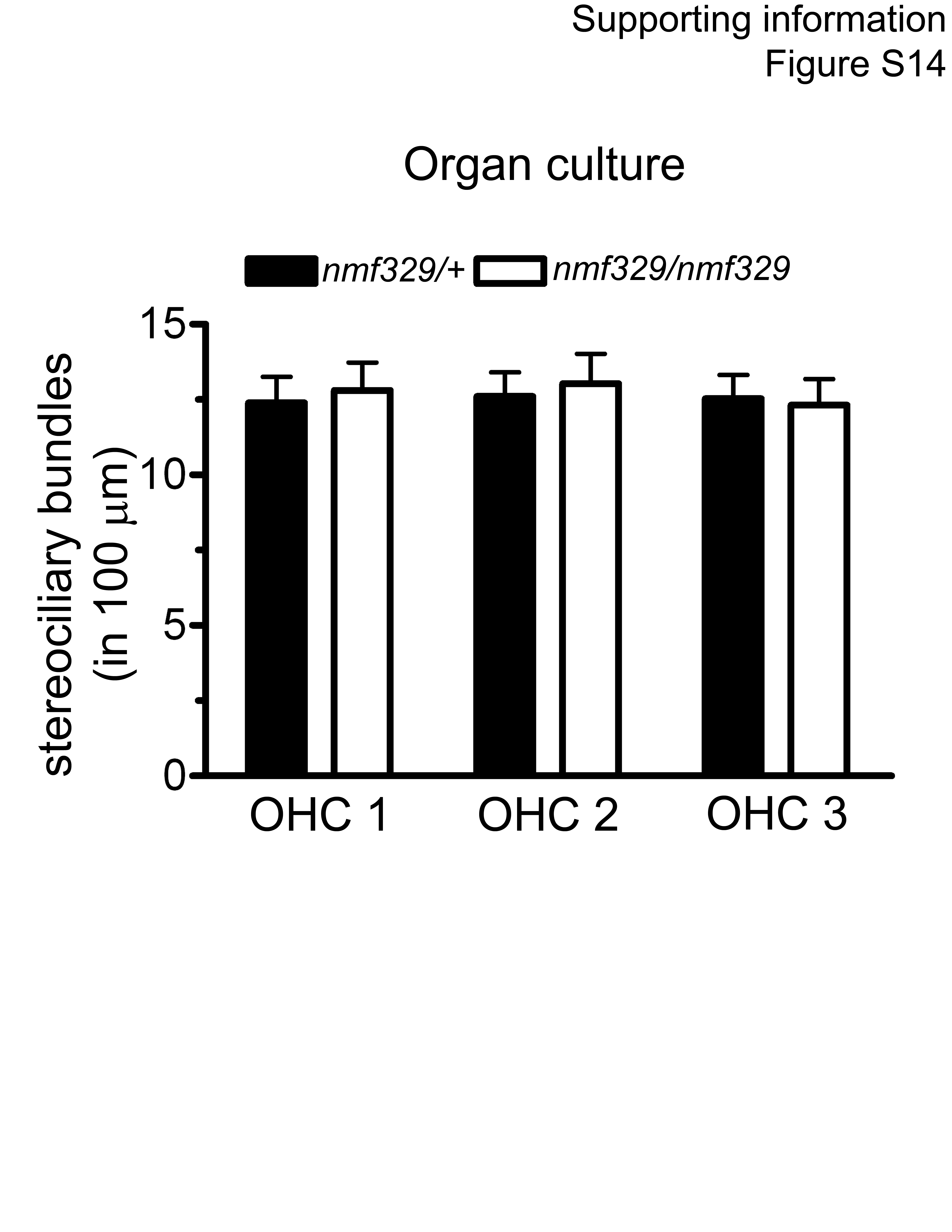

Supplement: Figure S14 — Counts of stereociliary bundles in cultured organ of Corti samples from heterozygous and homozygous nmf329 mice. Organ of Corti explants from nmf329/+ and nmf329/nmf329 mice (P5) were cultured for 9 days, and stereociliary bundles were counted in the first (OHC1), second (OHC2), and third (OHC3) rows of OHCs. Counts are normalized to 100 µm. Data are mean±SEM (n = 7 and 8 in the groups; unpaired t-test, p>0.05). (0.40 MB TIF) [file pgen.1000610.s014.tif]
